# Genome-Wide Analysis of Functional and Evolutionary Features of *Tele*-Enhancers

**DOI:** 10.1534/g3.114.010447

**Published:** 2014-02-04

**Authors:** Di Huang, Ivan Ovcharenko

**Affiliations:** Computational Biology Branch, National Center for Biotechnology Information, National Library of Medicine, National Institutes of Health, Bethesda, Maryland 20892

**Keywords:** enhancer, nucleotide divergence, single-nucleotide polymorphism, tissue specificity, transcription factor binding motif

## Abstract

We investigated sequence features of enhancers separated from their target gene by at least one intermediate gene/exon (named *tele*-enhancers in this study) and enhancers residing inside their target gene locus. In this study, we used whole genome enhancer maps and gene expression profiles to establish a large panel of *tele*-enhancers. By contrasting *tele*-enhancers to proximal enhancers targeting heart genes, we observed that heart *tele*-enhancers use unique regulatory mechanisms based on the cardiac transcription factors SRF, TEAD, and NKX-2.5, whereas proximal heart enhancers rely on GATA4 instead. A functional analysis showed that *tele*-enhancers preferentially regulate house-keeping genes and genes with a metabolic role during heart development. In addition, *tele*-enhancers are significantly more conserved than their proximal counterparts. Similar trends have been observed for non-heart tissues and cell types, suggesting that our findings represent general characteristics of *tele*-enhancers.

Enhancers, which are key to the precise regulation of spatiotemporal gene expression, often reside at a distance from their target genes (Maston *et al.* 2006) and function through long-range regulatory mechanisms (Glinskii *et al.* 2011; Lettice *et al.* 2003; Pomerantz *et al.* 2009). Although some enhancers are found in the proximity of the transcription start sites of their target gene, there is mounting evidence of distant enhancers incorporated into the structure of neighboring genes or looping over intermediate unaffected genes (Irimia *et al.* 2012; Lettice *et al.* 2003; Perry *et al.* 2010; Visser *et al.* 2012). As such, identifying target genes of enhancers, especially enhancers that regulate genes outside of the locus they reside in (which we dubbed *tele*-enhancers), poses a great challenge in the research area of gene regulation. To address this challenge, many have used evolutionary conservation of DNA sequences and focused on highly conserved enhancers (Clarke *et al.* 2012; Davidson *et al.* 2006; Engström *et al.* 2007; Kikuta *et al.* 2007). Assuming that enhancers and their target genes are located within a genomic regulatory block encompassing a set of neighboring genes and regulatory regions, regulatory relations between highly conserved enhancers and their target genes have been established, and transgenic models have been used to validate these regulatory relations (Dong *et al.* 2009; Kikuta *et al.* 2007; MacKenzie *et al.* 2004; Navratilova and Becker 2009; Sanyal *et al.* 2012). These studies have successfully identified enhancers and their distant target genes, which are instrumental for understanding the mechanisms and evolution of gene regulation. However, these investigations focused on individual *tele*-enhancers. Recently, with the advancement of sequencing techniques, such as chromatin conformation capture (3C, Hi-C) (Belton *et al.* 2012; Dixon *et al.* 2012) and chromatin interaction paired-end tagging (*i.e.*, ChIA-PET) (Li *et al.* 2010; Zhang *et al.* 2012), it became possible to experimentally characterize interactions between enhancers and their target genes. However, these experiments are notoriously difficult to run, and genome-wide data are available only in a few cell types. As a result, without the knowledge of a substantial number of *tele*-enhancers, we still have a scant genome-wide understanding of interactions between long-range enhancers and their target genes.

In this study, we binned heart enhancers into two groups—proximal and *tele*-enhancers—based on their genomic location relative to the nearest heart gene, and then systematically investigated the differences between proximal and distant regulatory mechanisms of transcriptional activation in the human heart. We observed that *tele*-enhancers have unique biological functions—regulating housekeeping genes and playing a role in basic biological processes. Next, we demonstrated that *tele*-enhancer sequences feature a distinguishable binding motif signature. Although both *tele*- and proximal heart enhancers are enriched for the binding motifs of several cardiac transcription factors (TFs) (such as MEF2A), *tele*-enhancer sequences are enriched for TEAD1 and NKX-2.5 binding motifs and are depleted of the GATA4 binding motif compared with proximal enhancers. We also demonstrated that *tele*-enhancers (1) are significantly more conserved than proximal enhancers, (2) display lower single-nucleotide polymorphism (SNP) density, and (3) are enriched for low derived allele frequency (DAF) SNPs—all suggesting that *tele*-enhancers have been and are currently evolving under a stronger negative selective pressure than their proximal counterparts. We then extended our analysis to other tissues and cell types (including fetal brain, fetal lung, HepG2, K526, HSMM, and H1-hESC cell lines), for which genome-wide enhancer maps were identified using different experimental methods, and observed similar evolutionary trends as well as sequence and functional features of *tele*-enhancers, suggestive of the general trends of this novel regulatory mechanism described by our study.

## Materials and Methods

### Heart enhancers and genes

**Figure 1:**
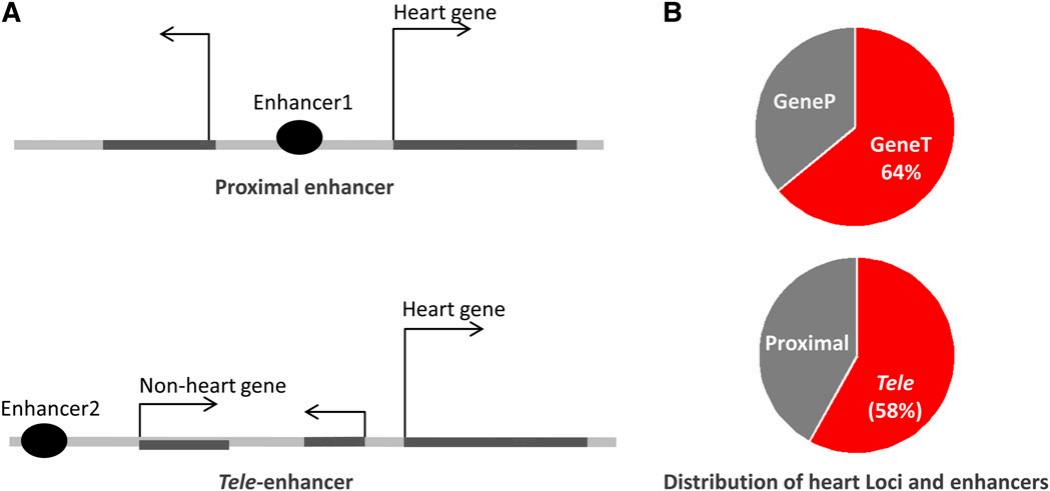
Proximal and *tele*-enhancers. (A) Schematic definition of two classes of enhancers. (B) Distribution of heart GeneTs and GenePs (top) and proximal and *tele*-enhancers (bottom).

We used the set of 5047 human heart enhancers identified by a p300 chromatin immunoprecipitation sequencing (ChIP-seq) study of fetal heart tissue (gestational week 16; Gene Expression Omnibus [GEO] data set series 32587) (May *et al.* 2012). We assigned each enhancer to the two nearest heart genes located within 500 kbs from the enhancer. As a result, an enhancer was assigned to at most two heart genes. After discarding enhancers not associated with any heart gene and heart genes not associated with any enhancer, we obtained 3391 enhancers linked to 1832 heart genes. We dubbed enhancers separated from their target genes by at least one nonheart gene or exon as *tele*-enhancers, and the enhancers located within the loci of heart genes as proximal enhancers (Figure 1). We also generated a set of control sequences. For each enhancer, two random noncoding human DNA sequences with matching length, guanine-cytosine (GC) content, and repeat density were retrieved.

### Enhancers and genes in other tissues and cell lines

Fetal brain enhancers were obtained from the H3K4me1 map established by ChIP-seq experiments carried out on the human fetal brain tissue (GEO dataset GSM706850, human fetal brain at day 122). To ensure that the nucleotide sequences used in this study are enhancers and not proximal promoters, we discarded sequences located within 3 kb of the transcription start site of any human gene. To obtain brain genes, we collected the genes annotated to the “brain development” or its child categories in the Gene Ontology (GO) database. We also used gene expression profiles (Su *et al.* 2004) and identified the top 20% of genes highly expressed in fetal brain with respect to other tissues and cell lines. After applying our method to these data, we assigned 7649 brain enhancers to 1957 brain genes (Supporting Information, Table S1).

Fetal lung enhancers also were obtained from the H3K4me1 map established by ChIP-seq experiments carried out on the human fetal lung tissue (GEO dataset GSM706853, human fetal lung at day 101). We similarly discarded sequences located within 3 kb of any transcription start site. To the end, we linked 5996 lung enhancers to 1716 lung genes (Table S1).

In cell lines including GM12878, 1H-hESC, HepG2, HSMM, HUVEC, K562, and NHEK, we used ChromHMM enhancer maps (Djebali *et al.* 2012; Ernst and Kellis 2012). Also, based on RNA-seq data reported in the ENCODE project (Djebali *et al.* 2012), we extracted the top 20% of genes highly expressed in a cell with respect to other cells as cell-specific genes. After applying our pipeline to these data (*i.e.*, cell-specific enhancers and genes), we identified ∼6000 *tele*-enhancers in each line (Table S2).

### Evaluation of enhancer-gene association

Enhancer-promoter maps have been previously constructed using the distribution of DNase I hypersensitive sites (DHS) across 79 distinct cell types, and the reported regulatory connections have been confirmed using 5C and chromatin immunoprecipitation paired-end sequencing data (Thurman *et al.* 2012).

Although the distal-DHS-promoter connection map was established on the basis of an extensive panel of cells, this map does not cover the entire regulatory-element-promoter landscape for all tissues and cell lines. For example, 1538 (of 3391) heart enhancers assigned to at least one heart gene could not be retrieved from this distal-DHS-promoter connection map. As such, we built the regulatory block for each gene based on distal-DHS-promoter connection map (denoted as DHS-based regulatory block in this study). Given a gene, the genomic boundaries of its DHS-based regulatory block were defined using the most distal DHS connected to that gene. Because the enhancers located within this block more likely regulate that gene than those located outside of this block, we evaluated whether enhancers reside within the DHS-based regulatory blocks of their associated genes. We estimated the fraction of the enhancers that fell into the regulatory blocks of their associated genes, and compared this fraction with a null distribution. The null distribution was established by randomly selecting genes. In detail, given an enhancer and its associated gene (the distance between them is D), we randomly selected a gene with the regulatory block of a similar length to the tested gene (*i.e.*, the length of the regulatory block of randomly selected genes was required to reside in the range of [0.9z, 1.1z] where z is the length of the regulatory block of the tested gene). Then, we checked whether the sequence located away from the selected gene at a distance of D fell into the regulatory block of the selected genes. For each enhancer, we repeated this process 20 times, and used the fraction of the sequences residing in the respective regulatory blocks as expected.

### Evolutionary synteny blocks (ESBs) and density of evolutionary breakpoints

ESBs are commonly used to identify boundaries of regulatory interactions, as regulatory elements and their target genes tend to reside within the same synteny block, if the regulatory mechanisms in question play an important functional role. Accordingly, the density of synteny breakpoints should be reduced between regulatory elements and their target genes.

We downloaded ESBs from the ECRBase database (Ovcharenko *et al.* 2005b) and evaluated the breakpoint density in the regions separating heart enhancers and their target genes. After that, the density of synteny breakpoints was compared with an expectation. Because the evolutionary breakpoints are not evenly distributed along genome (Carver and Stubbs 1997; Pevzner and Tesler 2003), we estimated the expected density of breakpoints “locally” instead of “globally.” That is, for each region spanning an enhancer and one of its target genes, we extended this region by 500 kb along both the upstream and downstream direction, and then excluded gene coding regions from the extended sequence. We used these extended regions as controls to estimate the expectation of breakpoint density.

### Control genes

To eliminate the potential impact of locus length on the functional analysis of genes, we generated control genes with a similar locus length to a given gene set. For a gene, we randomly selected five genes having a similar locus length. After repeating this process for all genes, we generated a control set for a tested gene set.

### Support vector machine (SVM) classification models and binding motifs enriched in enhancer sequences

To discriminate enhancer sequences from controls, we designed a computation system that included two main steps. In the first step, putative TF binding sites were mapped in the DNA sequence of enhancers and controls. For this, sequences were scanned using tfSearch (Ovcharenko *et al.* 2005a) with the position weight matrices from the TRANSFAC and JASPAR databases (Sandelin *et al.* 2004; Wingender *et al.* 2001). In the second step, TF binding site vectors of enhancers and controls were used to build a linear SVM (Cortes and Vapnik 1995) to discriminate between enhancers and controls. Given a training set of instances $\left\{{\text{x}}_{1},{\text{x}}_{2},\ldots ,x_{n}\right\}$ with associated labels $\left\{{\text{y}}_{1},{\text{y}}_{2},\ldots ,y_{n}\}\in \{-1,\;1\right\}$, a linear SVM $\text{y}=w^{T}\text{x}+\text{b}$ was built by solving the optimization problem $\text{min}\left(\frac{1}{2}w^{T}\text{w}+\text{C}\underset{i}{\sum }\varepsilon_{i}\right)$ subject to $y_{i}\left(w^{T}x_{i}+\text{b}\right)\geq 1-\varepsilon_{i}$ and $\varepsilon_{i}\geq 0$(Cortes and Vapnik 1995). In such linear SVM, a linear weight w_i_ is assigned to each TF binding site. A large positive w_i_ indicates a binding site that is strongly associated with the enhancers, whereas negative weights correspond to the binding sites associated with the control set. We ranked sites according to w_i_, *i.e.*, the positive association with the tested enhancer set.

### Functional analysis based on GO annotation

The enrichment of a GO functional category (C_i_) for a group of genes (G) was measured according to

$${\text{Pr}}_{\text{i}}=\underset{0<k<m}{\sum }\frac{\left(\begin{array}{c} M\\ k \end{array}\right)\left(\begin{array}{c} N-M\\ n-k \end{array}\right)}{\left(\begin{array}{c} N\\ n \end{array}\right)},$$

where m is the size of the overlap between C_i_ and G. M and N are the size of C_i_ and all genes, respectively. n is the size of G. To control the false-positive rate, we adopted the conservative Bonferroni multiple-testing correction strategy (Dunn 1961) to adjust Pr_i_ as aPr_i_ = KPr_i_, where K is the number of GO functional categories for the test.

### Nucleotide divergence and diversity

To evaluate nucleotide divergence, we aligned each enhancer sequence to chimpanzee and rhesus by using axt alignment files available from the UCSC Genome Browser (http://genome.ucsc.edu/index.html).

Nucleotide difference (d) of a DNA region was defined as the number of nucleotides different across species per site. According to the three-way alignment among human, chimpanzee, and macaque, we evaluated d specific to each species. Given a DNA region, d specific to human (dh) is measured as

$$\text{dh}=\frac{{\text{L}}_{\text{h}\neq (\text{c}=\text{r})}}{\text{La}},$$

where La is the length of three-way-alignable fragment along the given region, and L_h≠(c=r)_ is the number of aligned nucleotides where chimpanzee is equal to macaque, and human is the outlier with respect to chimpanzee and macaque. Nucleotide divergence specific to human (Dh) is then estimated by adjusting dh with Juke-Cantor correction for multiple hits (Jukes and Cantor 1969). In a similar way, the nucleotide divergence D specific to chimp (Dc) and specific to macaque (Dm) were measured.

To evaluate selection neutrality within the human lineage, we defined the neutrality index (NI), in which human-specific nucleotide divergence (*i.e.*, Dh) of enhancers is compared with non-human divergence (*i.e.*, Dr + Dc) with respect to the neutral reference.

$$\text{Neutrality Index}\;(\text{NI})=\frac{\text{Dh}/\text{Nh}}{\left(\text{Dr}+\text{Dc})/(\text{Nr}+\text{Nc}\right)},$$

where Nh, Nr, and Nc are the divergences specific to human, rhesus, and chimp. A large NI means a large nucleotide difference during the evaluation of human (Figure 5). Also, based on Dh, Dr + Dc, Nh and Nr + Nc, we carried out the McDonald-Kreitman test (MK test) (McDonald and Kreitman 1991) to estimate the significance of nucleotide divergence.

Nucleotide diversity (π) of a region is estimated as the number of SNPs per site in the alignable fraction of a region.

### Genes regulating mitochondrial biological processes

After going through the GO database (Ashburner *et al.* 2000), we collected 282 genes annotated to a mitochondrial biological process.

### Pseudogenes

Pseudogenes, dysfunctional gene homologs (Vanin 1985), were used as a neutral reference in this study. We downloaded pseudogenes from the Peseudogene.org database (Balasubramanian *et al.* 2009).

### Human-accelerated conserved noncoding sequences (HACNSs)

HACNSs used in this study were from Prabhakar *et al.* (2006).

## Results

### Identification of *Tele*-heart enhancers and their target genes

In this study, we used a conservative definition of *tele*-enhancers, requiring a presence of an intermediate gene (or an exon of an intermediate gene) between an enhancer and its target gene. To identify heart *tele*-enhancers, we first compiled a list of developmental heart genes according to gene expression profiles and gene annotation information. After ranking all human genes according to their expression level in the human heart relative to the other 78 tissues/cell types (Su *et al.* 2004) (http://hgdownload.cse.ucsc.edu/goldenPath/hg19/database/), we selected the top 20% (2342) of genes and observed that these genes are significantly enriched (1) in the heart development GO category (Ashburner, Ball *et al.* 2000) [*P* = 2 × 10^−11^, using DAVID (Jiao *et al.* 2012)], (2) in the neighborhood of p300 heart enhancers (hypergeometric distribution *P* = 4 × 10^−34^), and (3) in the heart disease genes reported by the GeneTests database (http://www.ncbi.nlm.nih.gov/gtr/; *P* = 4 × 10^−13^, Figure S1, and File S1), suggesting a significant association between these highly expressed genes and human heart development (see *Materials and Methods*). In addition, we combined these highly expressed genes with heart development genes annotated in GO (total = 348 genes), and then established a collection of 2430 distinct heart genes. The genomic landscape of these heart genes was superimposed onto 3391 heart development enhancers that were previously identified in a p300 ChIP-seq fetal human heart tissue experiment (Blow *et al.* 2010), and were located within no more than 500 kb from the transcription start site of a heart gene. Following a general rule postulated by Busser *et al.* (2012), we associated each heart enhancer with the two closest heart genes—one upstream and the other downstream of the enhancer. Enhancers separated from their nearest heart gene by at least one “nonheart” gene/exon were considered *tele*-enhancers (Figure 1A). In total, 3319 heart enhancers were associated with 1832 heart genes, out of which a large proportion of genes—1171 (64%)—had no proximal enhancers located on either the inside of the gene body, or in between the gene and its two flanking genes. These findings are in line with the report that proximity is not the governing rule for enhancers regulating genes, and less than 50% of expressed genes have proximal enhancers in several cell types (including embryonic stem cells) (Zhang *et al.* 2013). We hypothesized that the heart genes lacking proximal enhancers are likely regulated by *tele*-enhancers (Figure 1A). For simplicity, we dubbed a heart gene with at least one proximal heart enhancer GeneP, and a heart gene linked to *tele*-enhancer(s) only—GeneT. Although genes lacking proximal enhancers may be controlled through activation by regulatory elements other than enhancers, such as locus control regions (Li *et al.* 2002), most genome-wide gene regulation studies are confined to promoters and enhancers for simplicity and generalization (Gaszner and Felsenfeld 2006). Therefore, we assumed that GeneTs are primarily regulated by *tele*-enhancers. Also, before systemically characterizing *tele*- and proximal enhancers, we evaluated the reliability of the established assignments between genes and enhancers.

### Regulatory landscape of heart genes

**Figure 2:**
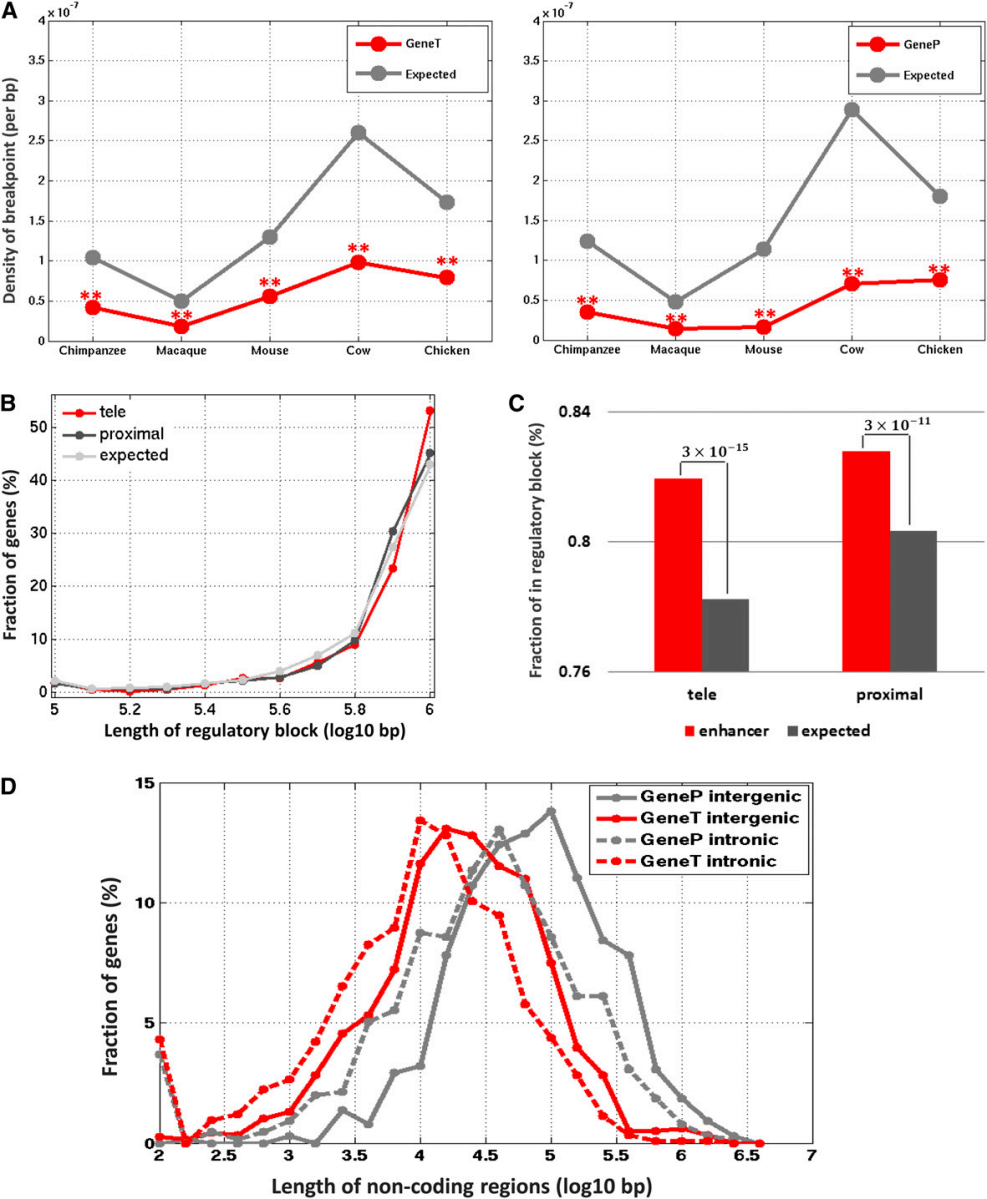
Heart genes and enhancers linked to them. (A) The average density of evolutionary breakpoints between enhancers and their target genes. A low density of breakpoint indicates a high probability of the enhancers and their target genes being located inside the same evolutionary synteny block. **Indicates that the corresponding density is significantly lower than expected, *i.e.*, *P*-value < 1 × 10^−5^. (B) Length distribution of DNase I hypersensitive sites (DHS)-based regulatory blocks of GeneTs and GenePs. (C) Fraction of enhancers residing within the DHS-based regulatory blocks of their associated genes. (D) Length distribution of noncoding regions (intergenic and intronic) of GeneTs and GenePs.

We used ESBs to confirm regulatory relationships between the *tele*-enhancers and their assigned target genes (see *Materials and Methods* for details). ESBs are a known characteristic of long-range regulatory interactions as the separation of critical regulatory elements from their target genes through chromosomal rearrangements is selected against (Ovcharenko *et al.* 2005b); ESBs have been successfully used to detect target genes of long-range enhancers (Dong *et al.* 2009; Kikuta *et al.* 2007; Navratilova and Becker 2009). Accordingly, we expected *tele*-enhancers and their target genes to reside within the same ESB more often than expected by chance. We used the defined ESBs based on the sequence alignments between human and chimpanzee, macaque, mouse, cow, and chicken (Ovcharenko *et al.* 2005b) and compared the density of the evolutionary breakpoints between enhancers and their target genes with the density expected in the neighborhood of enhancers (see *Materials and Methods*). Our comparative results showed that the genomic space separating enhancers from their target genes exhibited a significantly lower density of evolutionary breakpoints than would be expected. As such, the enhancers, either proximal or *tele*-enhancers, and their associated genes were predominantly located in the same ESB (*P* < 10^−5^ in all cases, Figure 2A).

Additional validation of the predicted enhancer-gene relationships was obtained using a genome-wide map of enhancer-promoter associations constructed based on a DNase I comparative profiling of the human genome (Thurman *et al.* 2012). Although the reported DNase I map of enhancer-promoter relationships is an approximation across a large panel of cell types, which does not necessarily represent a comprehensive collection of long-range regulatory activities in the heart, it estimates the extent of regulatory interactions across different gene loci. After defining a DHS-based regulatory block for each gene based on its most distal enhancers, we first noticed that GeneTs have longer DHS-based regulatory blocks as compared to GenePs (Wilcoxon rank sum *P* = 0.06, Figure 2B). Also, we computed the fraction of the enhancers located within the regulatory block of their associated genes, and demonstrated that 82% of *tele*-enhancers reside within the regulatory blocks of their associated genes, which was significantly higher than expected (binomial test *P* = 3 × 10^−15^, Figure 2C). Similarly, 83% of proximal enhancers were located within the regulatory blocks of their associated genes, which also was significantly higher than expected (binomial test *P* = 3 × 10^−11^, Figure 2C). These results further support the established regulatory relationship between the enhancers, either proximal or *tele*-enhancers, and their associated genes.

Next, we examined the size of intronic and intergenic regions of heart gene loci and noticed that, on average, GeneTs featured 3.0-fold shorter intronic regions than GenePs (Wilcoxon rank sum *P* = 2 × 10^−26^, Figure 2D). Similarly, the intergenic intervals flanking GeneTs were 2.78-fold shorter compared to GenePs (Wilcoxon rank sum *P* = 6 × 10^−64^, Figure 2D). It is likely that the small locus size of GeneTs predisposes these genes to the acquisition of *tele*-enhancers. Given the recent evolutionary nature of many heart enhancers (Blow *et al.* 2010), an alternative hypothesis of an intermediate gene insertion in between a heart enhancer and a GeneT is unlikely. From the evolutionary viewpoint, it has been found that the maintenance of gene function over a long evolutionary time leaves a selection signature of gene structure (Vinogradov 2006), and that tissue-specific genes might harbor long noncoding regions containing multiple regulatory regions, whereas widely expressed genes, lacking strong intron constraints, might have been subjected to selective pressure to reduce the length of noncoding regions (Eisenberg and Levanon 2003; Pozzoli *et al.* 2007). With this knowledge, and based on our finding that GeneTs showed significantly shorter intronic and intergenic spread than GenePs, we hypothesized that *tele*-enhancers and proximal enhancers, which respectively regulate GeneTs and GenePs, have different biological functions and undergo different evolutionary processes. To explore this hypothesis, we next examined functional and evolutionary features of *tele*- and proximal heart enhancers.

### *Tele*-enhancers regulate mitochondrial biological processes

**Figure 3:**
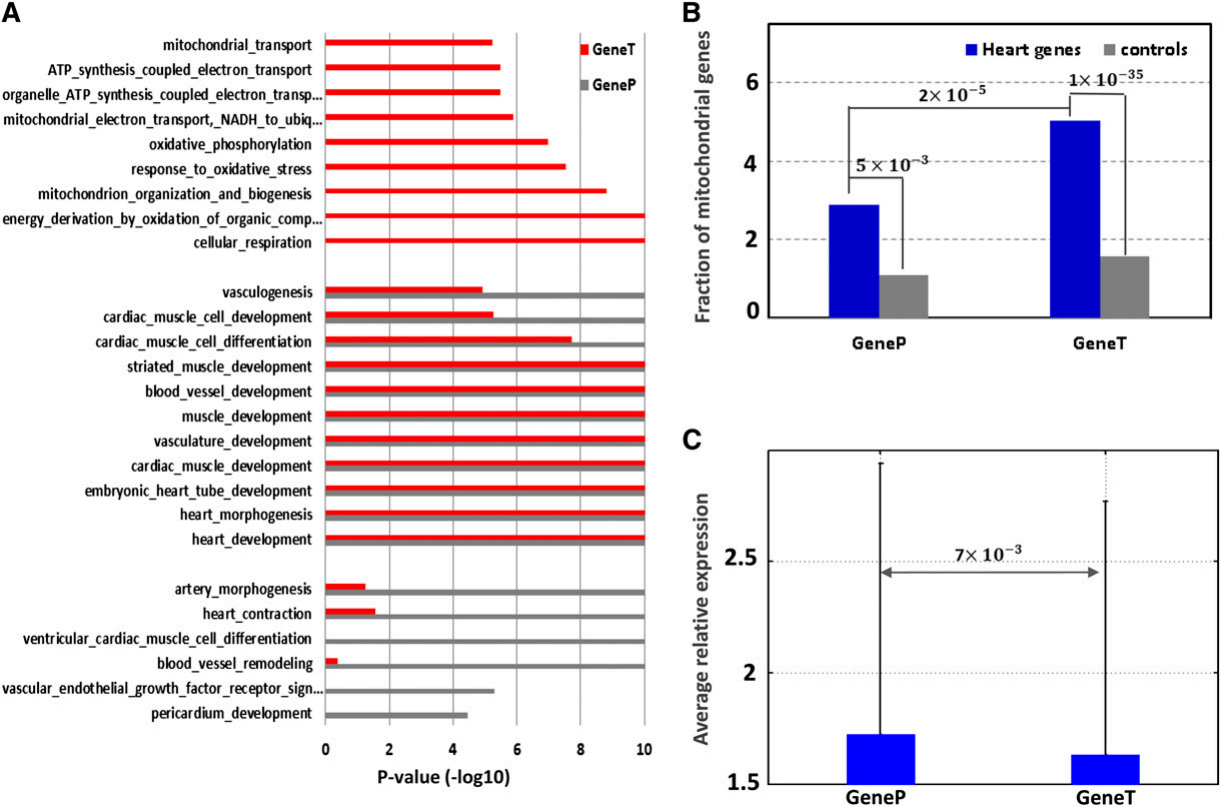
Function of *tele*- and proximal enhancers. (A) Functional analysis based on GO annotation. The enrichment is measured as the ratio of the fraction of the target genes having a tested function to the expectation with the matching locus length (*i.e.*, gene as well as its intergenic and intronic regions). The *P*-value is estimated using the binomial test. (B) Enrichment of mitochondrial genes among GeneTs and GenePs (corresponding to *tele*- and proximal enhancers, respectively). (C) The relative expression of GeneTs and GenePs—given a gene, a low relative expression indicates a weak specificity to heart.

We analyzed the function of the two heart enhancer groups—proximal and *tele*-enhancers—according to the GO function of their target genes (GenePs and GeneTs, respectively) (Ashburner *et al.* 2000). In this study, to account for the different locus lengths of GenePs and GeneTs (as discussed previously), which may cause a bias in a gene function analysis, we generated controls for GenePs and GeneTs separately by randomly selecting genes with similar length intergenic and intronic regions (see *Materials and Methods*), and compared GenePs/GeneTs with the respective control genes. As expected, both proximal and *tele*-enhancers play an important role in biological processes related to heart development, such as heart morphogenesis, cardiac muscle differentiation, etc. (multiple testing corrected binomial test *P* < 1 × 10^−3^, Figure 3A and Table S3). On the other hand, each heart enhancer group featured distinct functions—proximal enhancers were strongly associated with various processes related to heart development, whereas *tele*-enhancers were involved in basic metabolic functions. For example, of 18 genes associated with ventricular cardiac muscle cell differentiation (GO:0055012), 11 (*P* = 4 × 10^−4^) genes were GeneP, whereas only 3 were GeneT. Similarly, among 27 genes that fell into the category of artery morphogenesis (GO:0048844), 13 genes (*P* = 1 × 10^−7^) had proximal enhancers, while 7 genes were GeneT (Table S3). By contrast, 73 precursor metabolites and energy (GO:0006091) genes were categorized as GeneT, whereas only 28 as GenePs. We also identified genes taking part in the regulation of mitochondrial biological processes and observed that GeneTs, but not GenePs, were significantly enriched for those genes (Figure 3B, 2.9% of GenePs *vs.* 5.1% of GeneTs, binomial test *P* = 2 × 10^−5^). Our observation is supported by reports that regulation of mitochondrial processes is essential to heart development (Drenckhahn 2011; Goffart *et al.* 2004) and has been used as a therapeutic target in heart failure (Huss and Kelly 2005). Moreover, whereas both GeneTs and GenePs showed significant heart specificity compared with the expected (*i.e.*, respective control genes, Figure S2), GeneTs had significantly weaker heart specificity than GenePs (Figure 3C, the average of GeneTs and GenePs are 1.7 and 1.9, respectively, Wilcoxon rank-sum test *P* = 7 × 10^−3^). This further indicates that, compared with GenePs, GeneTs more likely partake in basic biological processes and display lower heart specificity.

We also looked into the function of bystander genes of *tele*-enhancers, *i.e.*, the genes which are located closer to *tele*-enhancers than the target genes of these enhancers, but are not regulated by these enhancers during heart development. The functional GO analysis indicated that these bystander genes were not significantly enriched for any biological function (data not shown), which further supports the established connections between *tele*-enhancers and their target genes.

Collectively, *tele*-enhancers and proximal enhancers have different biological functions. The former partake in basic biological processes, regulating mitochondrial biological processes, while the latter are more specific to heart development. Because *tele*-enhancers showed functions distinct from proximal enhancers, we hypothesized that *tele*-enhancers and proximal enhancers are involved in different transcriptional mechanisms and could be activated by different sets of TFs.

### *Tele*-enhancers feature distinguishable binding motif compositions

**Figure 4:**
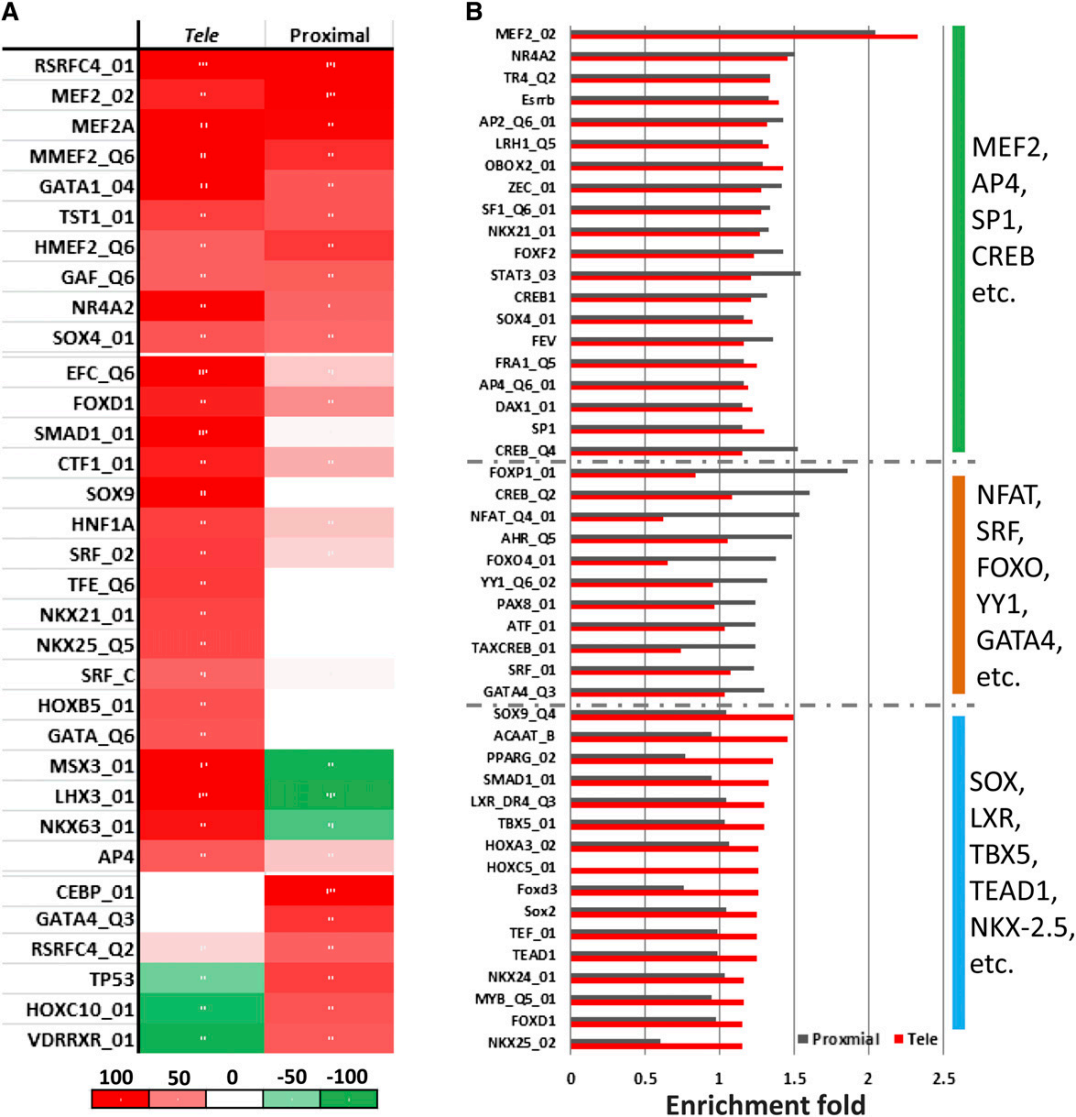
Association of TF binding motifs with different enhancer classes. (A) The weight of support vector machines built to discriminate enhancer sequences from controls. (B) Enrichment fold of transcription factor binding motifs in *tele*- and proximal enhancers.

We adapted a machine learning approach that has been previously used to identify motifs specific to heart enhancers (Narlikar *et al.* 2010). This method identifies specific sequence patterns for a set of non-coding sequences relying primarily on known TF binding motifs from the TRANSFAC and JASPAR databases (Matys *et al.* 2006; Stormo 2000). After mapping 981 vertebrate TF binding motifs onto enhancers and controls with similar GC content, repeat density and sequence length, we built two SVM classifiers with linear kernels based on the occurrence of TF binding motifs (see *Materials and Methods*)—one for *tele*-enhancers and another for proximal enhancers. TF motifs strongly associated with training enhancers received large positive weights. We selected the motifs with positive weights in either classifier and clustered these motifs based on the SVM weights, *i.e.*, the association with enhancer classes (Figure 4A). Only 26% (30 of 117) of TF binding motifs, in which cardiac TFs such as MEF2A were included, were shared between *tele*- and proximal enhancers. *Tele*-enhancers featured positive association with the binding motifs of well-known cardiac TFs SMAD1, SRF, NKX-2.5, and TEAD and no association with the binding motifs of the cardiac TF GATA4, which were specific to proximal enhancers (Figure 4A and Table S4).

We also investigated the enrichment of TF binding motifs in *tele*- and proximal enhancers and observed a striking difference in their motif composition (Figure 4B and Table S5). For example, the binding motifs of NKX-2.5, TBX5, and TEAD were strongly enriched in *tele*-enhancers but not in proximal enhancers, while the binding motifs of NFAT4 and GATA4 were specific to proximal enhancers. These results indicate existence of a set of cardiac TFs needed for the activation of both proximal enhancers and *tele*-enhancers, and a specific transcriptional modulation by different cardiac TFs within these two groups of enhancers. The differences in transcriptional mechanisms employed by proximal enhancers and *tele*-enhancers can potentially explain the difference in the biological function and expression patterns driven by these two groups of enhancers. The slight discrepancy between TF binding motif enrichment and the weights in linear SVMs (for example, the binding motif of TBX5 was exclusively enriched in *tele*-enhancer sequences while this motif has not received a large weight in either *tele*- or proximal enhancer SVMs) may be explained by the fact that enrichment evaluates TF binding motifs individually, while a linear SVM model estimates binding motifs collectively. Also, a high enrichment fold does not necessarily indicate that the abundance of a binding motif in enhancers is sufficient to distinguish enhancers from the rest of the non-coding genome. For example, the binding motif of TBX5, although exhibiting relatively high enrichment fold (1.3) in *tele*-enhancers, had a relatively low occurrence (0.03 per 1000 bps), which led to a small linear weight assigned to this motif in our SVM models.

### Different selective signatures of *Tele*- and proximal enhancers

It is known that the regulatory elements sharing the same cellular function and being activated in the same biological pathway evolve in concert and tend to have correlated selective signatures (Shapiro and Alm 2008). We next analyzed the selective constraints imposed on heart enhancer groups. Although developmental enhancers, as a whole, are under strong evolutionary constraint, the selective pressure imposed on enhancers varies greatly—phastCons, a conservation score, ranges from 0 (indicating no conservation) to 1 (perfect conservation) (Blow *et al.* 2010; Clarke *et al.* 2012). To analyze the selective constraint of enhancers, we used phastCons derived from 46 placental mammal sequence alignments (Siepel *et al.* 2005) and assigned the average phastCons along the tested sequence to that enhancer. Compared with proximal enhancers, *tele*-enhancers were more conserved (Figure S3): 15% of *tele*-enhancers had a phastCons greater than 0.2, whereas 13% of proximal enhancers reached this conservation level (binomial test, *P* = 7 × 10^−3^, *tele*- *vs.* proximal enhancers).

**Figure 5:**
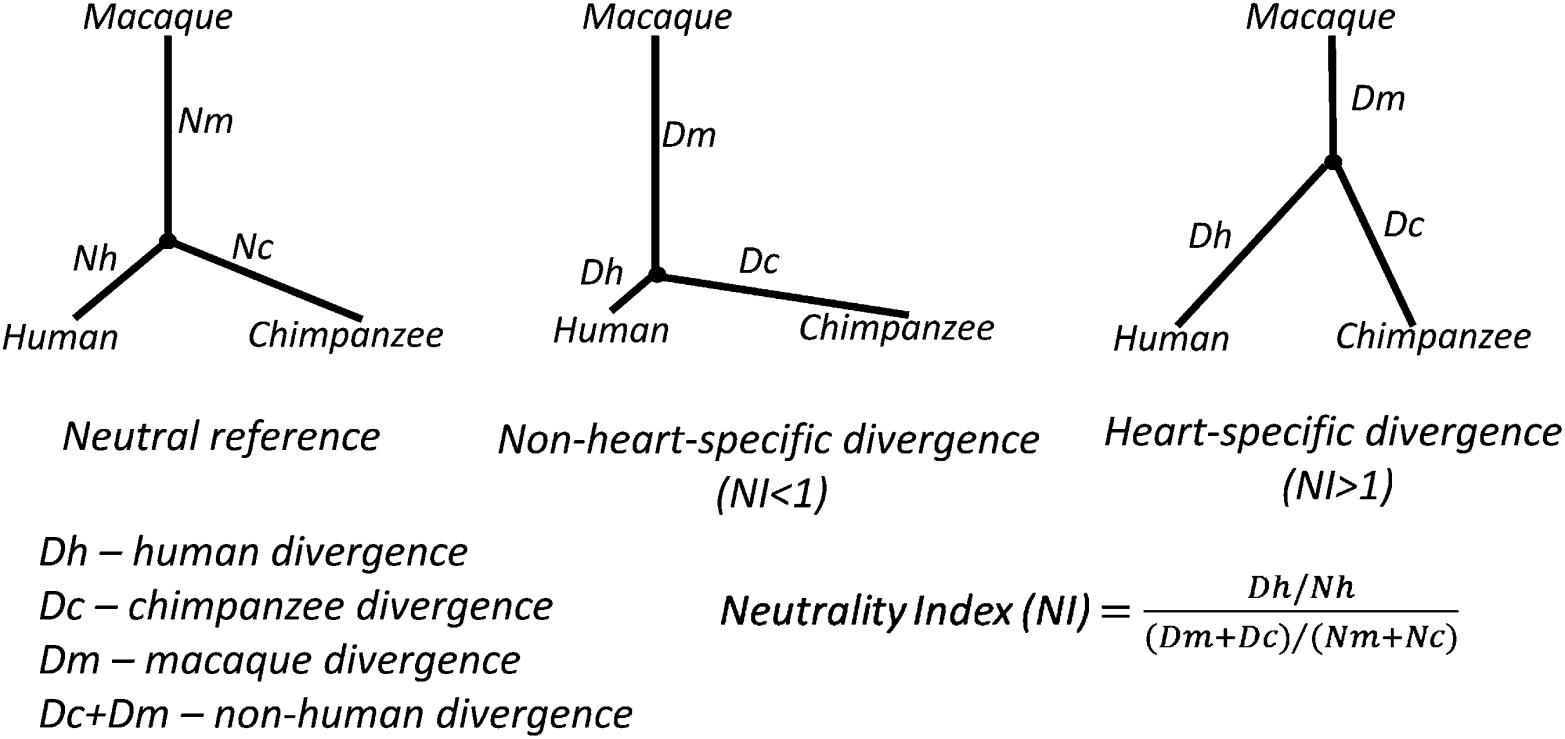
Schematic depiction of the human divergence, chimpanzee divergence, and macaque divergence based on three-way genome sequence alignments (see *Materials and Methods*). Through a comparison with neutral reference (pseudogenes in this study), the selective pressure acting on a tested region is measured using the neutrality index (NI). NI > 1 indicates positive selection, whereas NI < 1 corresponds to negative selection.

**Figure 6:**
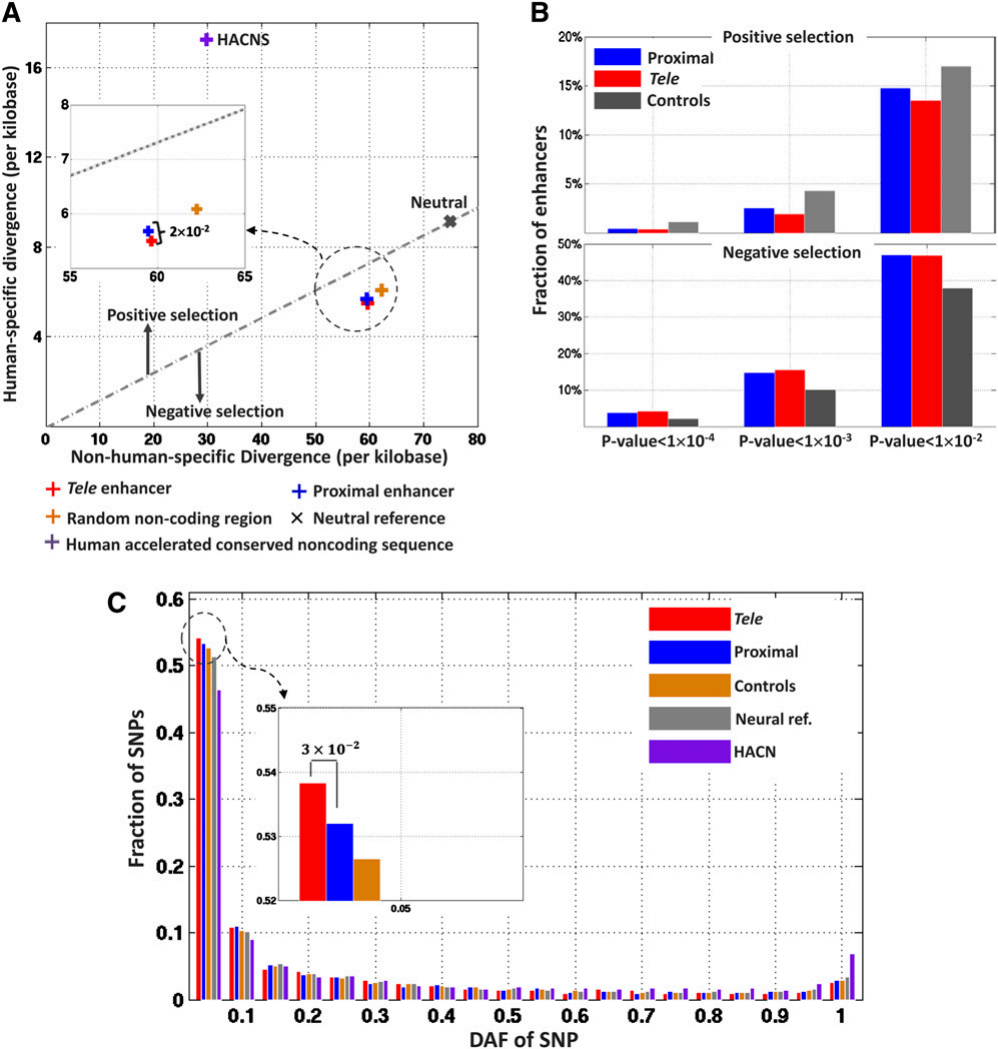
Divergence and diversity of enhancers across species. (A) Human divergence (y-axis) is plotted against nonhuman divergence (x-axis). HACNSs are human accelerated conserved noncoding sequences. (B) Fraction of enhancers under positive and negative selective pressure. (C) The derived allele frequency spectrum of SNPs in enhancers, control sequences, neutral reference, and HACNSs.

We next evaluated the selective pressure acting on enhancer sequences within the human lineage. After generating human-chimpanzee-macaque three-way alignments, we estimated the nucleotide divergence of enhancer sequence between any two species and evaluated the human-specific and non-human-specific divergence (Figure 5). The divergence rate of enhancer sequences was compared with the neutral divergence rate computed using pseudogenes. The comparative results showed that enhancers, either proximal or remote, had remarkably lower divergence levels than the control sequences (which were randomly generated along non-coding DNA with matched repeat density and GC content and the same length as enhancers) and HACNS (Figure 6A and Table 1). Next, we evaluated the selection constraints acting on sequences along the human lineage using NI. NI is defined in such a way that low NI (<1) and high NI (>1) indicate negative and positive selection during the human lineage evolution, respectively (Figure 5). According to the NI estimates, although both proximal enhancers and *tele*-enhancers featured negative selection, *tele*-enhancers with the average NI of 0.75 evolved under stronger negative selection constraints than proximal enhancers with the average NI of 0.77 (Table 1). Also, we evaluated the patterns of selective constraints in proximal enhancers and tele-enhancers using the MK test (McDonald and Kreitman 1991). Compared with the neutral reference, both proximal and *tele*-enhancers evolved under a significant negative selection pressure (*P* < 6 × 10^−23^, Fisher’s exact test, Table 1). Moreover, the negative selection pressure acting on *tele*-enhancers was significantly stronger compared with proximal enhancers (*P* = 2 × 10^−2^, Fisher’s exact test).

**Table 1:** Nucleotide divergence of heart enhancers, pseudogenes (neutral reference), control sequences, and HACNSs

Sequence Region	Divergence (per Kilobase)		NI	*P*-Value of Negative Selection	SNPs per kilobase
	Human-Specific	Nonhuman-Specific			
Enhancers					
Proximal	5.66	60.01	0.77	9 × 10^−50^	5.61
* Tele*	5.47	59.53	0.75	4 × 10^−59^	5.48
Others					
Neutral reference	9.14	74.9	1	–	9.65
Controls	6.42	62.34	0.84	3 × 10^−39^	5.94
HACNSs	17.24	29.75	4.75	1	3.68

HACNSs, human accelerated conserved noncoding sequences; NI, neutrality index; SNP, single-nucleotide polymorphism.

Next, we applied the MK test to evaluate the selective constraints of individual enhancers, and found that, compared to control sequences, both proximal and *tele*-enhancers tended to be under strong negative constraints, showing less cases of positive selection and more cases of purifying selection than controls (Figure 6B). On the other hand, compared with proximal enhancers, fewer *tele*-enhancers were under positive selection. For example, with the *P*-value cutoff of 1 × 10^−4^, proximal enhancers were more likely to be under positive selective pressure than *tele*-enhancers—2.5% proximal and 1.5% *tele*-enhancers evolved under positive selection (binomial test *P*-value = 4 × 10^−3^, proximal *vs.* *tele*-enhancers). In summary, the nucleotide divergence across different species indicates that *tele*-enhancers are under stronger negative selection than proximal enhancers.

Because of the difficulties in aligning sequences, nucleotide divergence is not as straightforward as SNPs in assessing the signature of selection of DNA regions. We therefore used SNP and allele frequency to evaluate the selective pressure acting on heart enhancers during modern human history. Using the genome variation data from the 1000 Genomes Project (1000 Genomes Project Consortium *et al.* 2010), we first observed that *tele*-enhancers harbor significantly less SNPs than proximal enhancers (binomial test *P* = 2 × 10^−3^, Table 1). Since a shift toward lower DAF indicates negative selection in the modern human history (Goode *et al.* 2010), we also analyzed DAF distribution of SNPs in heart enhancers. The results demonstrated that (1) *tele* and proximal enhancers represent a 2.4% and 1.7% excess of low-DAF SNPs (*i.e.*, SNPs having DAF < 0.05) compared with neutral reference, respectively (binomial test, *P* = 4 × 10^−13^ for *tele*-enhancers *vs.* neutral reference, and *P* = 3 × 10^−6^ for proximal enhancer *vs.* neutral reference, Figure 6C); (2) *tele*-enhancers are enriched in low-DAF SNPs compared with proximal counterpart (binomial test, *P* = 1 × 10^−2^, Figure 6C).

Also, we further partitioned *tele*-enhancers into subgroups—intronic and intergenic *tele*-enhancers with a premise that genomic locations of sequences may partially determine their evolutionary patterns (Halligan *et al.* 2004). We observed intronic *tele*-enhancers being under stronger selection constraint than intergenic tele-enhancers (Table S6), which is in accordance with the finding that functional intronic regions are under stronger selection pressure as compared to intergenic counterpart in mammals (Davidson *et al.* 2009). Furthermore, we compared intronic and intergenic *tele*-enhancers with their proximal counterparts, and observed that *tele*-enhancers are consistently more conserved than their proximal counterparts. In summary, *tele*-enhancers (1) are enriched for conserved sequences (those with >0.20 phastCons, Figure S3, where the weak significant *P*-value is partially due to the small sample pool used for statistical analysis); (2) show lower NIs; (3) have low SNP density; and (4) exhibit preference toward low-DAF SNPs (Table S6).

Finally, both the nucleotide divergence and SNP-based analyses lead to the same conclusion that although heart enhancers are under strong negative selection, the selection pressure acting on *tele*-enhancers (either intergenic or intronic) is even stronger than that on proximal counterparts. This is in accordance with the finding that many highly conserved enhancers are separated from their target genes by “bystander” genes (Akalin *et al.* 2009). Also, since *tele* and proximal enhancers share similar functions (for example, both these enhancer types play a role in heart development and heart morphogenesis), display common cardiac TF binding motifs (such as those of MEF2A) and exhibit common selective features (both of them are highly conserved as compared to controls), it could be expected that the evolutionary constraint difference between *tele* and proximal enhancers is only weakly significant (*P*-values are between 0.001 and 0.05) in almost all cases.

### *Tele*-enhancers from different tissues show consistent evolutionary and functional features

We extended the study to other tissues, including fetal brain and lung, in an effort to analyze the generalizability of our results. In each tissue, we collected potential enhancers based on ChIP-seq experiments targeting H3K4me1, an enhancer-associated histone mark, along with their target genes retrieved using gene expression profiles and Gene Ontology gene annotations. We observed that a large fraction of highly-expressed genes were GeneTs, *i.e.*, the genes with no proximal enhancers. For example, 47% of brain genes and 50% of lung genes were GeneTs (Table S1).

In the case of lung and brain enhancers, similarly to heart enhancers, *tele*-enhancers were strongly associated with the development of the corresponding tissue (multitest-corrected hypergeometric distribution *P*-values = 0, Table 2 and Table 3 as well as Table S7 and Table S8). In addition, *tele*-enhancers were more strongly associated with basic processes than proximal enhancers (multi-test corrected hypergeometric distribution P-values < 1 × 10^−3^). For example, among 23 genes regulating the response of nutrient level, which have influence on brain development (Georgieff 2007), six genes (26%) were brain GeneTs, whereas none were GenePs. Also lung GeneTs, not GenePs, were significantly enriched for the genes taking part in GTP metabolic and catabolic process [those genes play an essential role in structural patterning during lung development (Wan *et al.* 2013)]. Furthermore, GeneTs in brain displayed significantly lower relative expression than GenePs (the average of relative expressions of GeneTs and GenePs was 1.4 and 1.9; Wilcoxon rank sum test *P*-value = 3 × 10^−22^). Similarly, in lung, the relative expression of GeneTs was significantly lower than that of GenePs (the average of relative expression levels of GeneTs and GenePs was 1.9 and 2.1, respectively; *P*-value = 2 × 10^−4^). These suggest that GeneTs in brain and lung show relatively low tissue specificity in brain and lung, similar to GeneTs in heart.

**Table 2:** Functional analysis of proximal and *tele*- lung enhancers

GO ID	GO	Proximal		*Tele*	
		Enrichment Fold	*P*-Value	Enrichment Fold	*P*-Value
GO:0030323	Respiratory tube development	14.47	0	14.35	0
GO:0009725	Response to hormone stimulus	2.97	0	3.14	0
GO:0035295	Tube development	6.02	0	4.91	0
GO:0009719	Response to endogenous stimulus	2.85	0	2.91	0
GO:0009611	Response to wounding	2.83	0	2.49	0
GO:0030324	Lung development	15.12	0	14.59	0
GO:0014070	Response to organic cyclic substance	3.24	3.42E-06	2.74	1.24E-02
GO:0048598	Embryonic morphogenesis	3.48	0	2.19	1.28E-02
GO:0045596	Negative regulation of cell differentiation	4.34	0	2.32	1.42E-02
GO:0035239	Tube morphogenesis	4.34	4.44E-13	2.43	7.02E-02
GO:0030855	Epithelial cell differentiation	3.99	4.69E-10	2.52	1.82E-02
GO:0008283	Cell proliferation	2.6	2.41E-09	1.97	2.06E-02
GO:0055093	Response to hyperoxia	3.77	1	16.83	1.79E-07
GO:0007585	Respiratory gaseous exchange	2.2	1	12.11	2.09E-07
GO:0055082	Cellular chemical homeostasis	1.92	7.66E-02	2.63	1.48E-06
GO:0046039	GTP metabolic process	1.45	1	3.09	2.62E-06
GO:0006184	GTP catabolic process	1.44	1	3.11	3.61E-06
GO:0006873	Cellular ion homeostasis	1.97	4.87E-02	2.61	8.53E-06
GO:0045730	Respiratory burst	0	1	30.30	7.80E-06

GO, Gene Ontology; GTP, guanosine-5′-triphosphate.

**Table 3:** Functional analysis of proximal and *tele*- brain enhancers

GO ID	GO	Proximal		*tele*	
		Enrichment Fold	*P*-Value	Enrichment Fold	*P*-Value
GO:0030900	Forebrain development	14.21	0	9.05	0
GO:0021537	Telencephalon development	15.2	0	11.68	0
GO:0031175	Neurite development	5.42	0	3.66	0
GO:0007423	Sensory organ development	3.8	0	3.39	0
GO:0048812	Neurite morphogenesis	5.77	0	3.64	0
GO:0030182	Neuron differentiation	5.44	0	3.52	0
GO:0007417	Central nervous system development	8.04	0	8.76	0
GO:0021543	Pallium development	16.82	0	11.18	0
GO:0007420	Brain development	11.09	0	11.71	0
GO:0030902	Hindbrain development	12.72	0	12.53	0
GO:0001764	Neuron migration	7.81	0	4.40	1.16E−03
GO:0001843	Neural tube closure	8.65	8.66E−11	5.22	3.64E−03
GO:0043523	Regulation of neuron apoptosis	4.54	7.43E−10	3.11	5.82E−03
GO:0045665	Negative regulation of neuron differentiation	6.92	1.66E−05	5.80	7.99E−03
GO:0007611	Learning and/or memory	6.54	0	3.13	8.20E−03
GO:0021696	Cerebellar cortex morphogenesis	69.24	0	7.83	6.21E−02
GO:0021895	Cerebral cortex neuron differentiation	44.51	1.00E−10	8.70	1.21E−01
GO:0021680	Cerebellar Purkinje cell layer development	49.46	1.78E−12	8.70	1.21E−01
GO:0046907	Intracellular transport	1.58	1.36E−01	2.41	0
GO:0021854	Hypothalamus development	6.18	7.73E−01	46.98	5.86E−11
GO:0032107	Regulation of response to nutrient levels	0	1	31.32	6.22E−06

GO, Gene Ontology.

**Figure 7:**
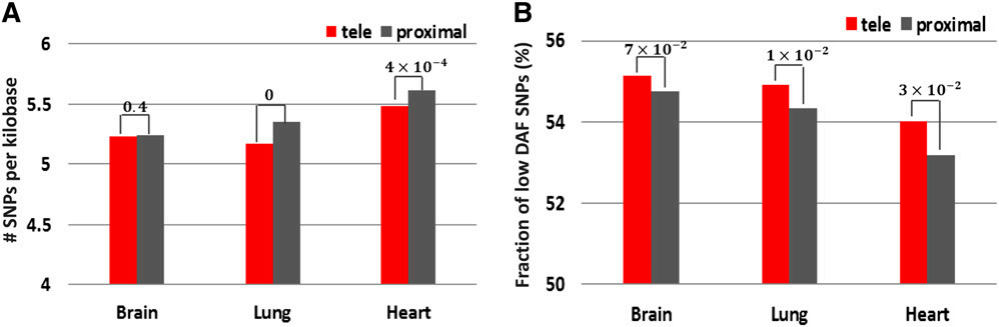
Single-nucleotide polymorphism (SNP)-based analysis of enhancers in three tissues (heart, brain, and lung). (A) Number of SNPs per kilobase of *tele*- and proximal enhancers. (B) Fraction of SNPs with low derived allele frequency (DAF) in *tele*- and proximal enhancers.

The analysis of sequence divergence of these enhancers indicated that the enhancers activated in different tissues evolve under negative selection but at a different degree of evolutionary constraint. With the lowest nucleotide divergence, brain enhancers were much more conserved than heart enhancers, which is consistent with previous reports (Blow *et al.* 2010) (Table 1 and Table 4). *Tele*-enhancers exhibited significant lower human-specific divergence than proximal enhancers (Fisher’s exact test *P*-value = 5 × 10^−3^ for brain, and *P*-value = 1 × 10^−2^ for lung, Table 4), suggesting stronger negative constraints imposed on *tele*-enhancers than on their proximal counterparts across all tested tissues. Additionally, SNP-based results consistently showed that *tele*-enhancers harbor less SNPs than proximal enhancers across all tested tissues. In heart and lung, the SNP density difference between *tele*- and proximal enhancers was significant (binomial test *P*-values < 4 × 10^−4^, Figure 7A), whereas this difference in brain was not significant. Also, *tele*-enhancers contained more low-DAF SNPs than proximal enhancers in all tested tissues (binomial test *P*-value = 3 × 10^−2^ for heart and lung, and *P*-value = 7 × 10^−2^ for brain, Figure 7B).

**Table 4:** Nucleotide divergence of lung and brain enhancers according to the human-chimpanzee-macaque sequence alignment

Sequence Region	Divergence (per kilobase)		Selection		
	Human-Specific	Nonhuman-Specific	NI	*P*-Value Against Neutral Ref.	*P*-Value of Proximal *vs.* *Tele*-
Brain enhancer					
Proximal	5.38	58.32	0.77	2 × 10^−48^	5 × 10^−3^
* Tele*-	5.38	57.02	0.75	8 × 10^−57^	
Lung enhancer					
Proximal	5.47	58.15	0.77	6 × 10^−50^	1 × 10^−2^
* Tele*-	5.30	57.35	0.75	3 × 10^−57^	

NI, neutrality index.

Taken together, *tele*-enhancers, although having different selective signatures across different tissues, have been consistently evolving under stronger negative constraints than proximal enhancers both during modern human history and during the separation of vertebrates, indicating that the observations we obtained from the study of heart enhancers are applicable to other tissues and represent a general trend in the evolution of proximal enhancers and *tele*-enhancers.

### *Tele*-enhancers from diverse cell types show consistent evolutionary and functional features

We also extended our study to a large panel of diverse cell types, including GM12878, 1H-hESC, HepG2, HSMM, HUVEC, K562, and NHEK, for which gene expression and ChromHMM enhancer maps have been previously reported (Djebali *et al.* 2012) (Ernst and Kellis 2012). We applied our pipeline to each of these cells and identified ∼6000 *tele*-enhancers in different cell lines (Table S2).

**Figure 8:**
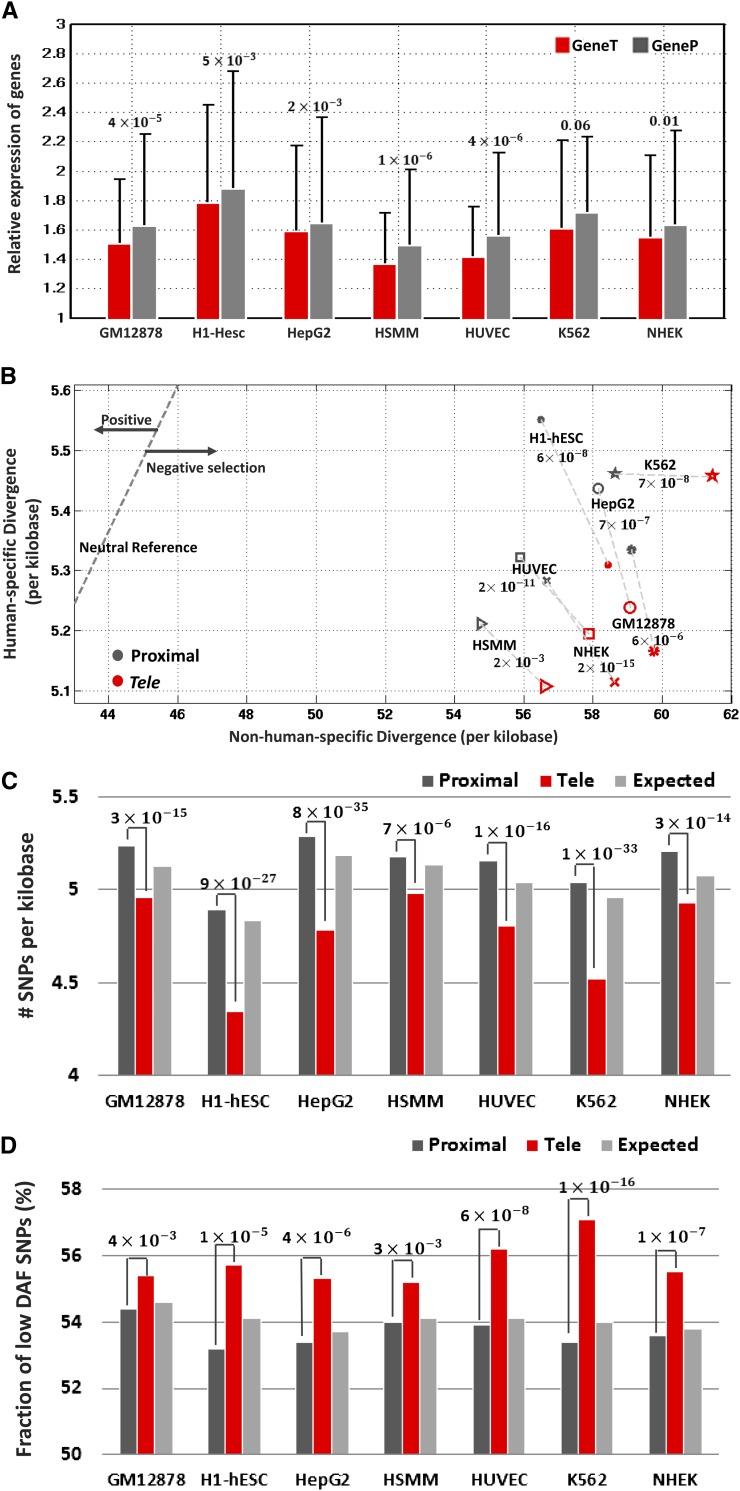
Features of *tele*-enhancers as compared to the respective proximal enhancers in seven cell types. (A) Relative expression of GeneTs and GenePs. (B) Nucleotide divergence of *tele*- and proximal enhancers. (C) Number of single-nucleotide polymorphisms (SNPs) per kilobase. All the differences between *tele*- and proximal enhancers are significant, *i.e.*, binomial test *P*-values < 7 × 10^−6^. (D) Fraction of SNPs having a low derived allele frequency (DAF < 0.05). All differences between *tele*- and proximal enhancers are significant, *i.e.*, *P*-values < 4 × 10^−3^.

In agreement with our heart *tele*-enhancer observations, GeneTs were found to be enriched in house-keeping genes as compared to the respective GenePs in all cell types. In all cells, GeneTs showed lower relative expression than GenePs. In all cases except K256, the relative-expression difference between GeneTs and GenePs was significant (Wilcoxon rank sum test *P*-values < 2 × 10^−3^, Figure 8A). These observations indicate that *tele*-enhancers likely play basic and fundamental biological roles independent of their cell-type specificity. Across all cell types, *tele*-enhancers featured remarkably lower human-specific divergence than their proximal counterparts (Fisher’s exact test *P*-values < 2 × 10^−3^) and neutrally evolving DNA (Figure 8B and Table S9), confirming the uniform nature of strong purifying selection acting on them. Moreover, *tele*-enhancers displayed a significantly lower SNP density across all cell types as compared to proximal enhancers (binomial test *P*-values < 7 × 10^−6^, Figure 8C). They also displayed a strong preference toward low-DAF SNPs—the fraction of low-DAF SNPs in *tele*-enhancers was lower than that in respective proximal enhancers in all cell types (binomial test *P*-values < 4 × 10^−3^, Figure 8D).

## Discussion

Understanding chromatin looping and its role in accurately positioning long-range enhancers into the immediate proximity of their target promoters has remained one of the most challenging problems of the postgenome era (Carter *et al.* 2002; Ernst 2012; Sanyal *et al.* 2012; Sexton *et al.* 2009; West and Fraser 2005). We observed that an underappreciated, large fraction of tissue-specific genes—64% of heart genes, 47% of brain genes, and 50% of lung genes—lack proximal enhancers and are being regulated by so-called *tele*-enhancers (enhancers that are separated from their target genes by at least one bystander gene or exon). This explicitly demonstrated that spatial proximity is not the mechanism by which enhancers activate their target genes and a large portion of enhancers might recognize their distant partners while “skipping” bystander genes (Zhang *et al.* 2013). However, our knowledge of the genome-wide distribution of *tele*-enhancers is very limited because previous studies of these requlatory elements focused on individual sequences instead of a genome-wide distribution (Dong *et al.* 2009; Navratilova and Becker 2009; Sanyal *et al.* 2012). To improve the understanding of *tele*-enhancers, we analyzed genome-wide enhancer maps established in a panel of tissues/cell types based on the activity of different biomarkers, such as the transcriptional coactivator p300, enhancer-associated histone mark H3K4me1, and a combination of regulatory-related chromatin signals. After assigning enhancers to their target genes on a genome-wide scale, we established the maps of *tele*-enhancers in three tissues (fetal heart, brain, and lung) and seven cell types (such as GM12878, H1-hESC, K562, etc.). We then compared *tele*-enhancers to proximal enhancers systematically and investigated functional, regulatory, and evolutionary mechanisms specific to *tele*-enhancers.

We demonstrated that the genes associated with heart *tele*-enhancers (GeneTs) partake in basic biological processes, showing lower heart specificity than genes associated with proximal enhancers (GeneP). Also, heart GeneTs have significantly shorter noncoding space in their neighborhood than GenePs. These findings are in line with the “selection for economy” model stating that widely-expressed gene loci are compact due to strong pressure for shortening non-coding regions and this might explain why they rely on *tele*-enhancers for transcription activation (Eisenberg and Levanon 2003; Pozzoli *et al.* 2007; Vinogradov 2006). Also, heart *tele*-enhancers have a TF binding motif signature distinct from proximal heart enhancers. For example, the binding motifs of TEAD and NKX-2.5 were overrepresented in *tele*-enhancers comparing to proximal enhancers whereas GATA4 displayed an opposite trend. These finding suggests that heart *tele*-enhancers, as compared to proximal counterparts, regulate distinct biological processes, and recruit different transcriptional activators.

We also measured the nucleotide divergence of heart enhancers between human, chimpanzee, and macaque and observed that heart *tele*-enhancers displayed low human-specific divergence. Also, heart *tele*-enhancers harbored less SNPs and more likely contained low-DAF SNPs compared with a neutral reference and their proximal counterparts. All these findings consistently suggested that heart *tele*-enhancers are under stronger negative selective pressure than proximal counterparts. Also *tele*- and proximal enhancers showed almost identical GC content, CpG site density, and ChIP-seq signal magnitude (Figure S4). After eliminating these possible confounding factors, we further ascertained that the genomic location of enhancers (*i.e.*, the position relative to potential target genes) was one of the determinant factors of functional and evolutionary signatures of heart enhancers.

We extended our study to fetal brain and lung, where enhancers were identified in ChIP-seq experiments targeting H3K4me1, and a panel of seven cell lines where enhancers were predicted according to chromatin signatures. The obtained results suggest that our results represent a common trend across different tissues and cell types.

How enhancers “travel” over intermediate regions and interact with remote core promoters to initiate transcription is one of the most enigmatic aspects of gene regulation (Kleinjan and van Heyningen 2005; Phillips and Corces 2009; Sexton *et al.* 2009). Our findings shed light on the interactions between remote enhancers and their targets, which are directly relevant to the development of future strategies for analyzing *tele*-enhancers and understanding their role in establishing complex gene regulatory landscapes of vertebrate genomes.

## Supplementary Material

Supporting Information
