# Supplementary material for: Genome-Wide Analysis of Functional and Evolutionary Features of Tele-Enhancers
Source: G3 (Bethesda). 2014 Feb 4;4(4):579–93. doi: 10.1534/g3.114.010447 (PMC4059231; doi:10.1534/g3.114.010447)
Supplement: Supporting Information [file supp_4_4_579__index.html]

Genome-Wide Analysis of Functional and Evolutionary Features of Tele-Enhancers — Supporting Information 

# Genome-Wide Analysis of Functional and Evolutionary Features of *Tele*-Enhancers

## Supporting Information for Huang and Ovcharenko, 2014

**Files in this Data Supplement:**

- Supporting Information - File S1, Figures S1-S4, and Tables S1-S9 (PDF, 1 MB)
- File S1 - Heart developmental genes (PDF, 194 KB)
- Figure S1 - Comparisons between AbsoluteRank and RelativeRank based on the enrichment of GO heart development genes, nearby genes of p300-bound heart enhancers and GeneTest heart disease genes. (PDF, 200 KB)
- Figure S2 - Comparisons of relative expression between heart genes and controls. (PDF, 328 KB)
- Figure S3 - Fraction of not-conserved and conserved enhancers in *tele* and proximal heart enhancers. (PDF, 313 KB)
- Figure S4 - Comparison of tele and proximal heart enhancers in terms of GC content, CpC site density and p300 ChIP-seq peak signal. (PDF, 239 KB)
- Table S1 - Distribution of GeneTs, GenePs, *tele* and proximal enhancers in fetal brain and lung. (PDF, 302 KB)
- Table S2 - Distribution of GeneTs, GenePs, *tele* and proximal enhancers in cell types. (PDF, 303 KB)
- Table S3 - GO biological processes associated with *tele* and proximal heart enhancers. (PDF, 364 KB)
- Table S4 - Weights of binding motifs in linear SVMs built for *tele* and proximal heart enhancers. (PDF, 442 KB)
- Table S5 - Enrichment Fold of TF binding motifs along *tele* and proximal heart enhancer sequences. (PDF, 473 KB)
- Table S6 - Nucleotide divergence and SNP distribution of intergenic/intronic *tele* and proximal heart enhancers (a) the value of divergence and SNP density; (b) p-values for all comparisons. (PDF, 421 KB)
- Table S7 - GO biological processes associated with *tele* and proximal brain enhancers. (PDF, 433 KB)
- Table S8 - GO biological processes associated with proximal and *tele* lung enhancers. (PDF, 322 KB)
- Table S9 - Nucleotide divergence (per kilobase) of *tele* and proximal enhancers in cell types. (PDF, 305 KB)
