# Supplementary material for: Genome-Wide Analysis of Functional and Evolutionary Features of Tele-Enhancers
Source: G3 (Bethesda). 2014 Feb 4;4(4):579–93. doi: 10.1534/g3.114.010447 (PMC4059231; doi:10.1534/g3.114.010447)
Supplement: Supporting Information [file supp_g3.114.010447_010447SI.pdf]

G3· Genes | Genomes | Genetics

Genome-Wide Analysis of Functional and Evolutional Features of *Tele*-Enhancers

Di Huang * and Ivan Ovcharenko *, 1

* Computational Biology Branch, National Center for Biotechnology Information, National Library of Medicine, National Institutes of Health, Bethesda, MD, 20892, USA
1 Corresponding author: 8600 Rockville Pike, Building 38A, Room 6S602, National Library of Medicine, National Institutes of Health, Bethesda, MD, 20892, USA. E-mail: ovcharen@nih.gov

DOI: 10.1534/g3.114.010447

File S1

Heart developmental genes

We compiled heart developmental genes from two sources – gene expression data and gene annotation. Using geome-wide gene expression profiles across 79 tissues/cells (Su, Wiltshire et al. 2004, see URLs) we ranked genes according to their absolute expression in heart (denated as AbsoluteRank), and to their relative expression in heart (denoted as RelativeRank). We evaluated top 20% genes in these gene lists using genes annotated to heart development and heart disease. Using the genes fallen into the category of heart development (GO:0007507) and its children categories, we collected 348 genes, and noticed that top 20% RelativeRank genes are most enriched for these heart genes (Figure S1). Similarly, we obtained a list of 71 heart-disease-related genes reported in GeneTests (see URLs), and observed that top 20% RelativeRank display highest enrichment for these genes (Figure S1). Finally we checked the distribution of the genes in the neighborhood of p300-bound heart enhancers (i.e., the enhancers used in this study) along ranked gene lists. More specifically, we collected all genes of which TSSs are in a distance of less-than-10k-bp to any p300-bound heart enhancers, Theinvestigation of distribution of these heart-enhancer-nearby genes show that these genes are enriched in top 20% RelativeRank. These evaluation results indicated that the top 20% RelativeRank gene list show the most association with heart development/disease, and thus were adopted in this study. After combining top 20% RelativeRank genes with the genes fallen into heart development category in GO, we finally obtained 2430 distinct heart developmental genes for further investigation.

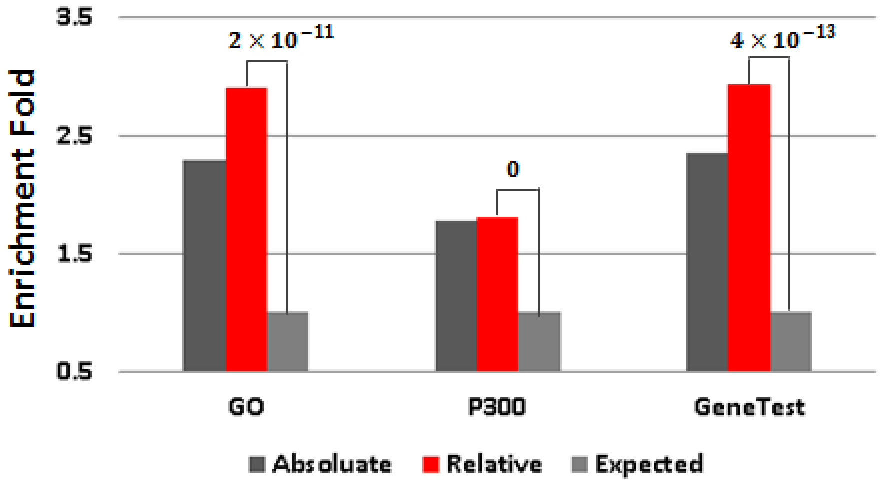

Figure S1 Comparisons between AbsoluteRank and RelativeRank based on the enrichment of GO heart development genes, nearby genes of p300-bound heart enhancers and GeneTest heart disease genes.

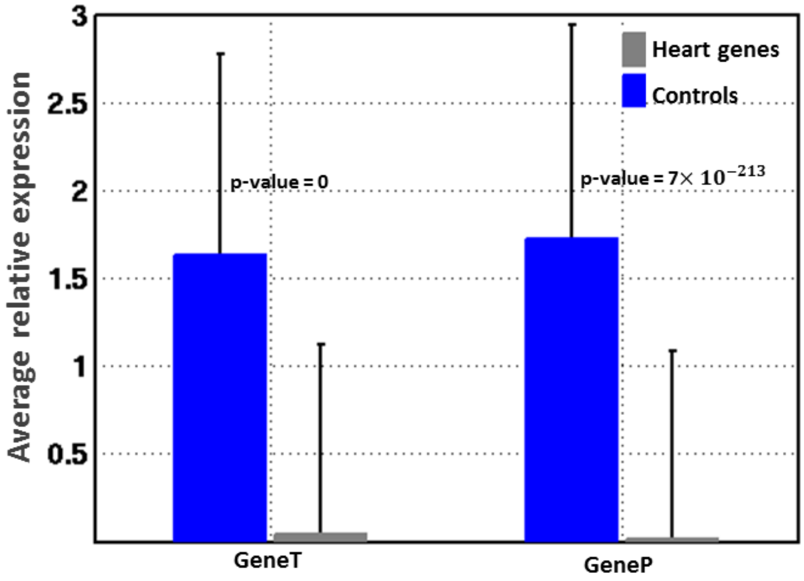

Figure S2 Comparisons of relative expression between heart genes and controls. Heart genes are grouped into GeneTs, which are associated with *tele*-enhancers, and GenePs, associated with proximal enhancers.

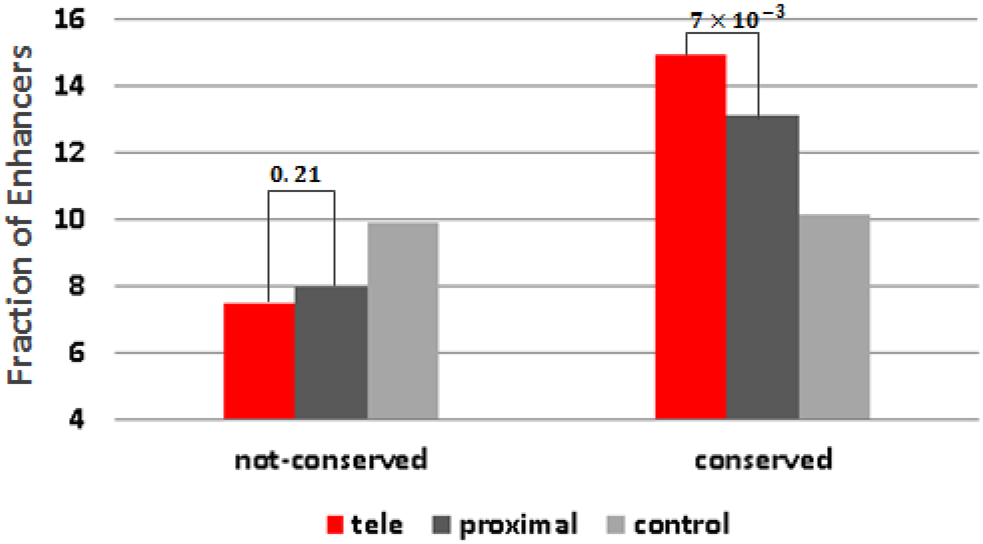

Figure S3 Fraction of not-conserved and conserved enhancers in *tele* and proximal heart enhancers. The conservation levels of sequences (either enhancers or controls) were estimated as the average of phastcon score. A sequence having an average phastcon score of < 0.05 was regarded as not-conserved, while an average phastcon score of >0.2 indicates a conserved sequence. The cutoff values for not-conserved and conserved were determined with reference to controls that are randomly-selected non-coding sequences having similar GC content, repeat density and same length to enhancers. After controls based on the conservation levels, we set the cutoff values so that 10% controls are conserved, and 10% controls are not conserved.

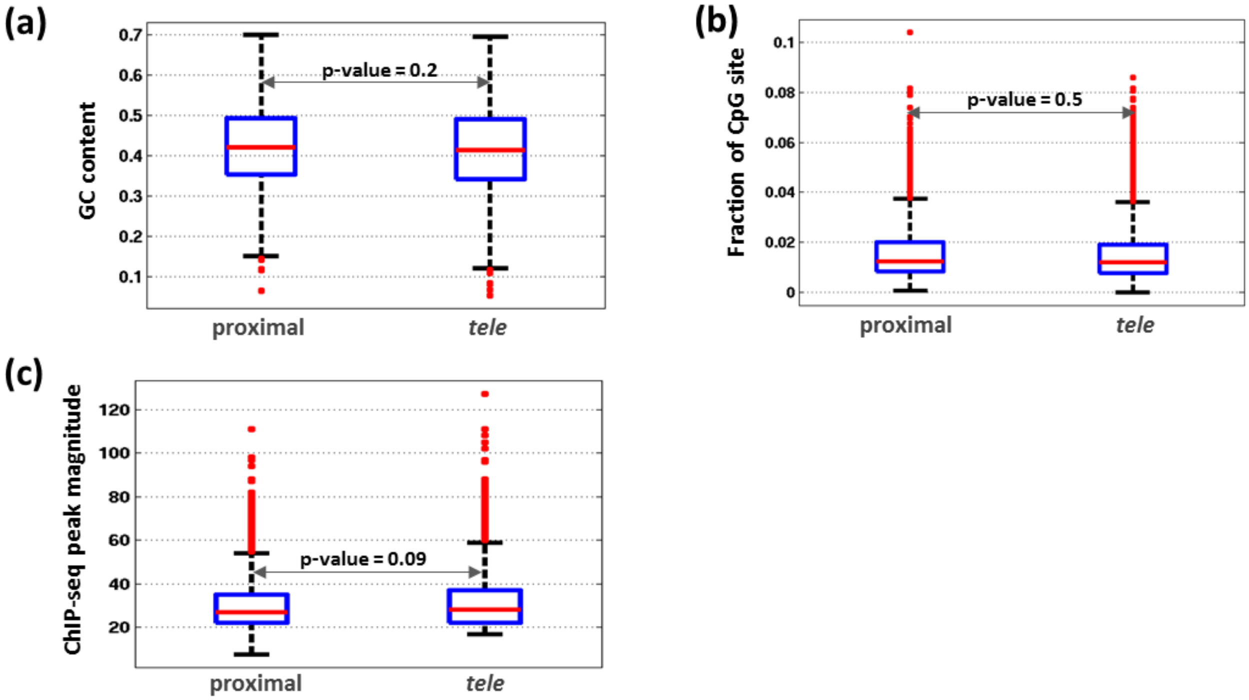

Figure S4 Comparison of tele and proximal heart enhancers in terms of GC content, CpC site density and p300 ChIP-seq peak signal.

Table S1 Distribution of GeneTs, GenePs, *tele* and proximal enhancers in fetal brain and lung.

	# Genes			# Enhancers			Fraction of multiple-tissue genes (%)			
	All	GeneT	GeneP	All	enT	enP	GeneA	GeneT	GeneP	pvalue (GeneT v.s. GeneP)
Brain	2689	927	1030	9604	4273	3376	28	30.1	26.3	0.12
Lung	2434	864	852	8934	3153	2816	58.3	60.3	55.8	0.09

Table S2 Distribution of GeneTs, GenePs, *tele* and proximal enhancers in cell types.

	#Gene	#GeneP	#GeneT	#enhancer	#proximal Enhancer	#tel-enhancer
GM12878	2398	1801	575	64090	21890	5730
H1-Hesc	2398	1250	1095	18076	4853	3332
HepG2	2398	1802	555	45107	17249	4261
HSMM	2398	1990	391	81275	30271	3642
HUVEC	2398	2068	312	79127	27915	3731
K562	2398	2022	344	69096	18351	4735
NHEK	2398	1996	388	86529	26526	5526

Table S3 GO biological processes associated with *tele* and proximal heart enhancers.

GOID	GO	Number of Genes	Proximal (associated with 661 genes)			Tele (associated with 1171 genes)		
			Number of Genes	Enrichment Fold	p-value	Number of Genes	Enrichment Fold	p-value
GO:0007507	heart development	297	120	13.13	0.00E+00	131	12.812562	0.00E+00
GO:0003007	heart morphogenesis	131	56	17.67	0.00E+00	55	11.46029	0.00E+00
GO:0035050	embryonic heart tube development	42	18	19.31	0.00E+00	20	15.97495	0.00E+00
GO:0048738	cardiac muscle development	85	45	24.13	0.00E+00	35	11.981213	0.00E+00
GO:0001944	vasculature development	343	89	7.96	0.00E+00	58	4.087708	0.00E+00
GO:0003012	muscle system process	185	42	7.77	0.00E+00	46	5.801429	0.00E+00
GO:0048729	tissue morphogenesis	326	72	5.29	0.00E+00	80	5.044721	0.00E+00
GO:0007517	muscle development	205	49	6.92	0.00E+00	48	4.694679	0.00E+00
GO:0009790	embryonic development	655	108	4.35	0.00E+00	112	2.710901	0.00E+00
GO:0035295	tube development	326	80	7.03	0.00E+00	70	3.727488	0.00E+00
GO:0001568	blood vessel development	322	84	8.5	0.00E+00	56	4.399658	0.00E+00
GO:0006936	muscle contraction	160	38	7.84	0.00E+00	43	6.061084	0.00E+00
GO:0014706	striated muscle development	178	57	9.26	0.00E+00	49	6.179783	0.00E+00
GO:0035239	tube morphogenesis	211	54	6.74	0.00E+00	47	4.416604	0.00E+00
GO:0001701	in utero embryonic development	259	43	4.8	0.00E+00	55	4.118542	0.00E+00
GO:0002009	morphogenesis of an epithelium	242	57	5.36	0.00E+00	49	4.048824	8.88E-13
GO:0007389	pattern specification process	301	54	3.86	0.00E+00	57	3.502201	1.78E-12
GO:0055010	ventricular cardiac muscle morphogenesis	28	10	53.63	8.88E-13	15	17.971819	4.44E-12
GO:0035051	cardiac cell differentiation	62	34	36.47	0.00E+00	20	10.649967	6.22E-12
GO:0048598	embryonic morphogenesis	345	69	5.44	0.00E+00	65	3.054035	1.15E-11
GO:0001947	heart looping	32	14	18.77	1.60E-11	16	15.335952	7.99E-12
GO:0042692	muscle cell differentiation	184	56	8.12	0.00E+00	38	4.441815	7.37E-11
GO:0048514	blood vessel morphogenesis	271	72	8.78	0.00E+00	43	3.816238	3.10E-10
GO:0055008	cardiac muscle morphogensis	36	13	17.43	3.34E-10	20	19.16994	0.00E+00
GO:0048644	muscle morphogenesis	45	15	11.49	3.41E-09	20	13.692814	0.00E+00
GO:0055007	cardiac muscle cell differentiation	45	29	38.88	0.00E+00	15	10.269611	1.91E-08
GO:0055001	muscle cell development	82	33	16.09	0.00E+00	21	5.920129	1.32E-07
GO:0001501	skeletal development	304	56	4.48	0.00E+00	46	2.755679	2.57E-06
GO:0051146	striated muscle cell differentiation	122	45	12.7	0.00E+00	25	4.279005	2.64E-06
GO:0055006	cardiac cell development	26	16	85.81	0.00E+00	9	14.377455	5.58E-06
GO:0055013	cardiac muscle cell development	26	16	85.81	0.00E+00	9	14.377455	5.58E-06
GO:0001570	vasculogenesis	55	22	19.66	0.00E+00	15	6.535207	1.22E-05
GO:0055002	striated muscle cell development	63	25	16.76	0.00E+00	15	5.990606	4.06E-05

GO:0001569	patterning of blood vessels	26	12	8.04	2.67E-05	8	12.77996	9.03E-05
GO:0003013	circulatory system process	222	41	6.28	0.00E+00	32	2.323629	2.64E-02
GO:0008015	blood circulation	217	39	5.98	0.00E+00	32	2.323629	2.64E-02
GO:0003015	heart process	30	12	64.36	0.00E+00	8	6.38998	2.66E-02
GO:0060047	heart contraction	26	11	58.99	0.00E+00	8	6.38998	2.66E-02
GO:0048844	artery morphogenesis	27	13	69.72	0.00E+00	7	6.709479	5.44E-02
GO:0006937	regulation of muscle contraction	77	19	7.84	6.44E-09	16	3.06719	1.26E-01
GO:0043433	negative regulation of transcription factor activity	85	17	5.07	6.60E-05	16	3.06719	1.26E-01
GO:0048771	tissue remodeling	73	15	8.04	6.15E-07	12	3.594364	1.84E-01
GO:0001974	blood vessel remodeling	27	9	48.27	4.44E-11	6	5.750982	4.26E-01
GO:0060070	Wnt receptor signaling pathway through beta-catenin	64	16	7.8	2.79E-07	12	3.382931	3.34E-01
GO:0016202	regulation of striated muscle development	73	22	10.73	4.44E-13	12	3.026833	9.75E-01
GO:0043535	regulation of blood vessel endothelial cell migration	30	11	9.83	1.06E-05	5	3.993738	1.00E+00
GO:0060039	pericardium development	12	8	14.3	3.41E-05	4	6.38998	1.00E+00
GO:0048010	vascular endothelial growth factor receptor signaling pathway	22	6	32.18	5.13E-06	5	4.792485	1.00E+00
GO:0048660	regulation of smooth muscle cell proliferation	64	13	7.75	1.22E-05	8	2.255287	1.00E+00
GO:0043552	positive regulation of phosphoinositide 3-kinase activity	24	6	32.18	5.13E-06	0	0	1.00E+00
GO:0031668	cellular response to extracellular stimulus	150	26	7.75	1.33E-12	19	1.979505	1.00E+00
GO:0043269	regulation of ion transport	278	35	3.35	1.26E-06	32	1.870238	1.00E+00
GO:0006942	regulation of striated muscle contraction	30	10	17.88	9.35E-08	6	3.19499	1.00E+00
GO:0055024	regulation of cardiac muscle development	27	12	64.36	0.00E+00	6	4.792485	1.00E+00
GO:0055012	ventricular cardiac muscle cell differentiation	18	11	58.99	0.00E+00	3	2.875491	1.00E+00
GO:0055015	ventricular cardiac muscle cell development	13	7	37.54	1.18E-07	3	4.792485	1.00E+00
GO:0048705	skeletal morphogenesis	142	30	7	0.00E+00	18	2.331479	1.00E+00
GO:0045333	cellular respiration	126	11	2.46	1.00E+00	40	7.099978	0.00E+00
GO:0015980	energy derivation by oxidation of organic compounds	258	22	2.11	1.00E+00	56	4.969985	0.00E+00
GO:0052548	regulation of endopeptidase activity	240	17	1.6	1.00E+00	47	3.633013	1.45E-10
GO:0052547	regulation of peptidase activity	252	18	1.69	1.00E+00	48	3.539066	2.07E-10
GO:0002697	regulation of immune effector process	165	13	2.49	1.00E+00	36	4.313237	7.77E-10
GO:0007005	mitochondrion organization	149	14	2.35	1.00E+00	36	4.208036	1.59E-09

	and biogenesis							
GO:0031099	regeneration	109	9	1.61	1.00E+00	26	5.663846	2.68E-09
GO:0006818	hydrogen transport	97	8	2.52	1.00E+00	26	5.191859	1.90E-08
GO:0015992	proton transport	96	8	2.52	1.00E+00	26	5.191859	1.90E-08
GO:0006979	response to oxidative stress	203	16	2.32	1.00E+00	38	3.642289	2.88E-08
GO:0006119	oxidative phosphorylation	47	4	2.68	1.00E+00	18	7.188728	1.03E-07
GO:0032374	regulation of cholesterol transport	30	0	0	1.00E+00	7	33.547395	2.90E-07
GO:0032371	regulation of sterol transport	30	0	0	1.00E+00	7	33.547395	2.90E-07
GO:0006120	mitochondrial electron transport, NADH to ubiquinone	32	3	2.3	1.00E+00	14	8.386849	1.30E-06
GO:0042775	organelle ATP synthesis coupled electron transport	41	4	2.68	1.00E+00	15	7.188728	3.23E-06
GO:0042773	ATP synthesis coupled electron transport	41	4	2.68	1.00E+00	15	7.188728	3.23E-06
GO:0006839	mitochondrial transport	76	6	1.61	1.00E+00	20	5.044721	5.86E-06
GO:0051186	cofactor metabolic process	219	15	1.71	1.00E+00	38	2.845538	2.78E-05
GO:0043122	regulation of I-kappaB kinase/NF-kappaB cascade	140	9	1.34	1.00E+00	26	3.664842	3.25E-05
GO:0030811	regulation of nucleotide catabolic process	273	23	1.62	1.00E+00	38	2.80176	4.18E-05
GO:0033121	regulation of purine nucleotide catabolic process	273	23	1.62	1.00E+00	38	2.80176	4.18E-05
GO:0031334	positive regulation of protein complex assembly	71	7	1.5	1.00E+00	16	5.477126	5.47E-05
GO:0002673	regulation of acute inflammatory response	36	0	0	1.00E+00	10	9.58497	5.93E-05
GO:0002757	immune response-activating signal transduction	171	9	1.79	1.00E+00	30	3.19499	6.68E-05
GO:0051348	negative regulation of transferase activity	138	16	2	1.00E+00	25	3.63067	7.13E-05
GO:0009308	amine metabolic process	457	27	1.34	1.00E+00	63	2.126243	7.17E-05

Table S4   Weights of binding motifs in linear SVMs built for *tele* and proximal heart enhancers.

Rank	Motif	*Tele*	Proximal
1	RSRFC4 01	229	141
2	MEF2 02	85	174
3	MEF2A	101	98
4	MMEF2 Q6	110	82
5	GATA1 04	101	65
6	TST1 01	75	67
7	HMEF2 Q6	63	78
8	GAF Q6	62	63
9	NR4A2	99	60
10	SOX4 01	67	58
11	NF1 Q6	104	48
12	MEF2 05	126	20
13	SOX Q6	88	55
14	HOXB4 01	136	0
15	EFC Q6	112	21
16	FOXD1	87	45
17	DBX2 01	124	0
18	SMAD1 01	118	3
19	CTF1 01	87	32
20	GBX2 01	119	0
21	P53 05	84	32
22	SOX9	110	0
23	Gata1	66	39
24	ERR1 Q2	82	20
25	HNF1A	75	23
26	PXR Q2	62	35
27	SOX9 Q4	79	16
28	SRF 02	76	16
29	AP4 Q5	65	22
30	TCF11MAFG 01	85	0
31	RAX 01	77	6
32	CLOCKBMAL Q6	90	-9
33	BRCA 01	81	0
34	IRX5 01	110	-32
35	TFE Q6	76	0
36	BRN4 01	76	0
37	NKX21 01	72	0
38	NKX25 Q5	72	0
39	DOBOX5 01	69	0
40	HOXB5 01	68	0
41	DMRT1 01	71	-5
42	LMX1B 01	66	0
43	GATA Q6	65	0
44	SRF C	61	4
45	CREB 01	64	0
46	MTATA B	76	-23
47	HEB Q6	85	-38
48	HOXA10 01	88	-45
49	FOXP3 Q4	67	-30
50	Mafb	69	-37
51	NKX63 01	93	-71
52	CART1 02	65	-55
53	MSX3 01	106	-96
54	LHX3 01	145	-212
55	AP1 Q4 01	41	183
56	Sox2	0	180

57	STAT6 01	0	175
58	POU1F1 Q6	19	148
59	MEF2 Q6 01	48	115
60	NCX 02	44	117
61	AP1 Q6 01	-1	151
62	CEBP 01	0	142
63	FOXO4 01	0	138
64	HNF1B 01	26	109
65	NKX52 01	24	96
66	SRY	0	118
67	MRG2 01	0	110
68	STAT3 03	25	85
69	PITX3 Q2	0	105
70	LHX5 01	-62	165
71	HOXB7 01	40	62
72	HNF4 Q6 01	38	63
73	PAX1 B	0	99
74	MYB Q3	18	81
75	REL	0	96
76	EVI1 05	-21	116
77	MAX	0	95
78	FXR Q2	0	93
79	MEF2 04	-14	105
80	OTX Q1	21	67
81	HNF3A 01	16	69
82	BRCA1	0	84
83	MYOGNF1 01	6	78
84	HNF1 Q6	16	66
85	VSX1 01	-25	107
86	PITX3 01	0	81
87	SRF 01	0	80
88	DLX1 01	16	63
89	Nobox	0	79
90	GATA4 Q3	0	78
91	RSRFC4 Q2	16	62
92	IPF1 Q4	14	64
93	CIZ 01	0	77
94	NRF2 Q4	-3	80
95	HSF2 02	0	75
96	BEN 02	0	75
97	CREB Q2	14	61
98	NKX3A 02	10	63
99	DLX7 01	0	73
100	CDX1 01	0	71
101	FEV	0	71
102	FOXD3 01	0	69
103	USF C	-3	72
104	MTF1 01	0	67
105	IRF2 01	0	62
106	TITF1 Q3	0	61
107	LEF1TCF1 Q4	0	60
108	TR4 Q2	-9	62
109	SHOX2 01	-45	94
110	IPF1 Q4 01	-49	95
111	Lhx3	-22	66
112	LHX3 02	-119	161
113	CEBPGAMMA Q6	-87	130
114	SP1 Q4 01	-53	83

115	TP53	-57	75
116	HOXC10 01	-86	67
117	VDRRXR 01	-96	65

Table S5 Enrichment Fold of TF binding motifs along *tele* and proximal heart enhancer sequences.

Motif	*Tele*	Proximal
MEF2 02	2.33	2.04
MEF2 03	2.12	1.95
RSRFC4 01	2.17	1.95
MEF2A	2.67	1.86
RSRFC4 Q2	2.46	1.84
MEF2 05	2.6	1.71
HMEF2 Q6	2.62	1.6
MEF2 Q6 01	3.12	1.59
NR4A2	1.45	1.5
ESR2	1.44	1.4
PXR Q2	1.51	1.39
RORA2 01	1.38	1.4
AMEF2 Q6	1.89	1.35
ELK1 02	1.35	1.42
TR4 Q2	1.34	1.34
Esrrb	1.39	1.33
ERR2 01	1.32	1.33
AP2 Q6 01	1.32	1.42
NFKB Q6 01	1.31	1.42
NFKAPPAB 01	1.33	1.31
BEN 02	1.33	1.3
DBP Q6	1.4	1.3
PAX3 01	1.52	1.3
NF1 Q6	1.6	1.29
LRH1 Q5	1.33	1.29
OBOX2 01	1.42	1.29
ZEC 01	1.28	1.41
SF1 Q6 01	1.28	1.34
NF-kappaB	1.28	1.28
NUR77 Q5	1.28	1.43
RORA Q4	1.34	1.28
Zfx	1.28	1.34
NKX21 01	1.27	1.33
E2F1DP1 01	1.27	1.33
MEF2 04	1.68	1.27
Mafb	1.31	1.27
RORA 1	1.27	1.27
AP1 C	1.27	1.26
PADS C	1.25	1.28
POLY C	1.25	1.36
FREAC2 01	1.32	1.25
ERR1 Q2	1.24	1.66
UF1H3BETA Q6	1.24	1.25
MTF1 02	1.33	1.24
EFC Q6	1.6	1.24
ER Q6	1.31	1.23
FOXF2	1.23	1.42
AP2GAMMA 01	1.22	1.24
GABPA	1.22	1.26
SP1 Q2 01	1.3	1.21
AP1 Q4 01	1.25	1.21
STAT3 03	1.21	1.54
CREB1	1.21	1.32
ZFP281 01	1.21	1.29
OBOX3 01	1.24	1.21
TEL2 Q6	1.21	1.25

CETS1P54 01	1.2	1.24
RFX Q6	1.24	1.2
SREBP2 Q6	1.2	1.25
KLF15 Q2	1.36	1.2
MIF1 01	1.2	1.23
TST1 01	1.37	1.2
RORA 2	1.2	1.33
AHR 01	1.2	1.37
MYB Q3	1.19	1.24
MAZR 01	1.23	1.19
MMEF2 Q6	1.56	1.19
SP1 Q4 01	1.19	1.23
ZF5 B	1.19	1.33
E2F1 Q6 01	1.29	1.18
EGR3 01	1.18	1.29
USF Q6	1.26	1.18
Egr1	1.19	1.18
AP1 01	1.18	1.38
IK2 01	1.21	1.18
P53 03	1.18	1.19
E2F1 Q3	1.18	1.28
ER Q6 02	1.21	1.18
HOXB8 01	1.18	1.29
AP2ALPHA 01	1.17	1.25
TFAP2A	1.17	1.25
IK1 01	1.17	1.18
WT1 Q6	1.38	1.17
P300 01	1.17	1.31
E2F1 Q4	1.22	1.17
PU1 Q6	1.17	1.18
E2F4DP2 01	1.17	1.26
Stat3	1.17	1.25
NFKB C	1.17	1.2
P53 01	1.17	1.42
ARNT 01	1.2	1.17
SP1 Q6 01	1.17	1.17
RUNX1	1.17	1.49
XVENT1 01	1.17	1.32
SRF	1.36	1.17
CBF 02	1.17	1.16
GATA1 04	1.16	1.27
AP1FJ Q2	1.16	1.4
SP1SP3 Q4	1.16	1.26
FREAC4 01	1.16	1.26
SF1 Q6	1.16	1.33
GR Q6 01	1.16	1.46
MECP2 02	1.17	1.16
SOX4 01	1.22	1.16
FEV	1.16	1.36
FRA1 Q5	1.25	1.16
AP4 Q6 01	1.19	1.16
PITX3 01	1.21	1.16
MAX	1.16	1.19
DAX1 01	1.22	1.15
SP1	1.3	1.15
CREB Q4	1.15	1.52
EGR2 01	1.15	1.2
FOXP1 01	0.84	1.86

CDX Q5	0.96	1.82
PXRRXR 02	0.86	1.78
HOXA9 01	0.65	1.62
EAR2 Q2	1.01	1.61
CREB Q2	1.08	1.6
DMRT7 01	0.91	1.54
NFAT Q4 01	0.62	1.53
MYOGNF1 01	1.07	1.52
FOXO1 Q5	0.86	1.48
AHR Q5	1.05	1.48
KAISO 01	0.93	1.48
ZNF219 01	1.1	1.48
IRF2 01	1.02	1.45
Spz1	1.05	1.43
OSF2 Q6	0.88	1.43
CDC5 01	0.69	1.41
DMRT3 01	0	1.41
PBX1 01	0.63	1.41
TBX15 02	0.61	1.4
HNF1B 01	1.02	1.4
CREBP1 Q2	1.04	1.39
FXR Q3	0.99	1.39
IRF7 01	1.04	1.39
Pax2	1.04	1.38
Pax5	1	1.38
PAX6 01	0.97	1.38
HOXB9 01	0.71	1.38
STAT1	1.07	1.38
CEBP C	0.98	1.37
AHRARNT 02	1.05	1.37
FOXO4 01	0.65	1.37
E2F1 Q6	1.07	1.36
GR 01	0.96	1.36
NFKAPPAB65 01	0.99	1.36
ATF1 Q6	0.98	1.34
CREB Q4 01	1.06	1.34
DMRT5 01	0.72	1.33
HOMEZ 01	0.97	1.33
PAX9 B	0.9	1.33
HBP1 Q2	0.77	1.33
INSM1	1.03	1.33
CREB Q2 01	0.98	1.33
PEBP Q6	0.78	1.33
IRF2	1.01	1.32
PITX2 Q2	1.02	1.32
AR 03	1.08	1.32
YY1 Q6 02	0.95	1.32
MAF Q6	1.06	1.32
IRF Q6 01	1.05	1.31
TITF1 Q3	0.88	1.31
GEN INI2 B	0.93	1.31
Nr2e3	0.91	1.3
ZTA Q2	1.06	1.3
DEAF1 01	0.95	1.29
GATA2 01	0.88	1.29
AFP1 Q6	0.52	1.29
CREB 02	0.87	1.29
STAT1 02	0.78	1.29

TAL1BETAE47 01	0.92	1.28
NFKB Q6	0.99	1.28
HNF1 02	0.64	1.28
NRF1 Q6	1.04	1.27
CBF 01	0.96	1.27
E12 Q6	0.74	1.27
RELA	1.04	1.27
GRE C	0.83	1.27
SP3 Q3	1.09	1.26
AML Q6	1.06	1.26
CP2 01	0.96	1.26
HNF4ALPHA Q6	0.97	1.26
CEBPGAMMA Q6	0.95	1.26
COUP 01	0.89	1.25
NR2F1	0.89	1.25
YY1 Q6	1.01	1.25
SRF Q5 02	0.9	1.25
PAX8 01	0.96	1.24
ATF 01	1.03	1.24
TAXCREB 01	0.74	1.24
SRF 01	0.97	1.23
MTF1 Q4	1.08	1.23
IPF1 Q4 01	0.67	1.23
CEBPA	0.89	1.23
CREBATF Q6	1.08	1.23
RNF96 01	1.06	1.23
PAX4 04	0.85	1.23
CMYB 01	1.08	1.22
HNF3A 01	0.94	1.22
ETS Q4	1.04	1.22
SZF11 01	1.05	1.22
ETS Q6	1.08	1.21
REST 01	1.01	1.21
STAT1 05	1	1.2
WHN B	1.09	1.2
ETS1 B	1.04	1.2
IK3 01	0.95	1.19
NKX25 03	0.68	1.19
AP2ALPHA 03	1.04	1.19
FOXO3	1	1.19
FPM315 01	1.07	1.19
AP2ALPHA 02	0.98	1.19
TCF4 Q5	0.66	1.18
LEF1 Q2 01	0.97	1.18
BARHL2 01	0.5	1.18
E4F1 Q6	0.9	1.18
PAX6 Q2	0.94	1.18
ZF5 01	0.86	1.18
LPOLYA B	0.9	1.18
NHLH1	1.04	1.18
ZBED6 01	1.08	1.17
PUR1 Q4	1.04	1.17
STAT6 01	1	1.17
HMBOX1 01	0.53	1.17
DLX7 01	0.95	1.17
FOXI1	0.96	1.17
GATA1 02	1.05	1.16
TCF3 01	0.78	1.16

TCF11 01	1.04	1.16
HNF3ALPHA Q6	0.96	1.16
IRF1 01	1.04	1.16
SRF 02	1.08	1.16
HIC1 03	0.96	1.16
AP1 Q4	1.07	1.16
HNF3 Q6	1	1.16
NURR1 Q3	1.07	1.15
SEF1 C	1.03	1.15
En1	0.8	1.15
PAX6 02	1.66	0
BRCA 01	1.57	0.75
HEB Q6	1.54	0.95
CART1 02	1.51	0
XFD1 01	1.5	0
HMGIY 01	1.5	0.9
SOX9 Q4	1.49	1.04
GATA1 05	1.49	0.94
CART1 03	1.49	0
ACAAT B	1.45	0.94
EN1 02	1.44	0
PAX7 01	1.43	0
GATA Q6	1.42	0.89
RBPJK Q4	1.41	0.97
ESR1	1.41	1.07
TFE Q6	1.4	1.08
HOXD3 01	1.39	1.03
RFX1 02	1.37	1.02
GATA2 03	1.36	0.56
ATF6 01	1.36	1.08
PPARG 02	1.36	0.77
USF 01	1.36	1.08
KROX Q6	1.35	1.04
AP4 Q5	1.34	1.02
NF1 Q6 01	1.34	1.01
NR3C1	1.33	1.09
NANOG 02	1.33	0.88
E2F1DP2 01	1.33	1.01
SMAD1 01	1.33	0.94
CDP 03	1.32	0.9
TCF11MAFG 01	1.32	0.89
HNF4 01 B	1.31	1.05
E2F 02	1.31	1.07
LXR DR4 Q3	1.3	1.04
SPIB 01	1.3	0.98
TBX5 01	1.3	1.03
CDP 04	1.29	0.88
FREAC7 01	1.29	0
IPF1 03	1.29	0.98
PPARG	1.28	1
GATA2 02	1.28	0.87
RREB1 01	1.28	1.02
T3R 01	1.28	0.96
GAF Q6	1.28	0.93
DOBOX4 01	1.28	0.66
MOX1 01	1.28	0.63
PAX4 02	1.28	0.69
HOXC4 01	1.28	0

GFI1B 01	1.28	0.89
PAX4 03	1.27	0.99
TATA 01	1.27	0.88
TBP	1.27	0.88
MEF3 B	1.26	0.97
HOXA3 02	1.26	1.06
PMX2B 01	1.26	0.65
HOXC5 01	1.26	0
ALX4 02	1.26	0.53
GATA3 02	1.26	0.79
PARP Q3	1.26	0.93
Foxd3	1.26	0.76
Sox2	1.25	1.04
TEF 01	1.25	0.98
TEAD1	1.25	0.98

Table S6 Nucleotide divergence and SNP distribution of intergenic/intronic *tele* and proximal heart enhancers (a) the value of divergence and SNP density; (b) p-values for all comparisons.

(a)

	divergence per 1000 bp		NI	total divergence		#SNP per 1000bp	Fraction of low DAF
	human	non-human					
Intronic							
tele	5.2995	58.7827	0.73	12889	142965	5.34	0.5432
proximal	5.4781	58.1271	0.77	7650	81173	5.41	0.5363
Intergenic							
tele	5.8521	61.1104	0.77	6355	66362	5.63	0.5238
proximal	5.882	62.2086	0.78	6818	72108	5.92	0.5273

(b)

NI			
	tele	proximal	pvalue
Intronic	0.73	0.77	1.00E-02
Intergenic	0.77	0.78	0.75
pvalue	1.00E-03	0.86	
SNP density			
	tele	proximal	pvalue
Intronic	5.34	5.41	3.00E-02
Intergenic	5.63	5.92	9.00E-06
pvalue	5.00E-04	8.00E-16	
Fraction of low DAF			
	tele	proximal	pvalue
Intronic	0.5432	0.5363	1.00E-01
Intergenic	0.5238	0.5273	0.43
Pvalue	5.00E-03	0.07	

Table S7 GO biological processes associated with *tele* and proximal brain enhancers.

GOID	GO	#genes	Proximal			Tele		
			#genes	Enrichment Fold	p-value	#genes	Enrichment Fold	p-value
GO:0030900	forebrain development	228	135	14.21	0.00E+00	78	9.048112	0.00E+00
GO:0021537	telencephalon development	135	83	15.2	0.00E+00	47	11.683002	0.00E+00
GO:0031175	neurite development	471	148	5.42	0.00E+00	80	3.663203	0.00E+00
GO:0007423	sensory organ development	326	76	3.8	0.00E+00	63	3.390351	0.00E+00
GO:0048812	neurite morphogenesis	420	140	5.77	0.00E+00	69	3.638227	0.00E+00
GO:0009792	embryonic development ending in birth or egg hatching	406	79	2.92	0.00E+00	71	3.33896	0.00E+00
GO:0030182	neuron differentiation	658	209	5.44	0.00E+00	114	3.521227	0.00E+00
GO:0007417	central nervous system development	558	239	8.04	0.00E+00	183	8.763962	0.00E+00
GO:0021543	pallium development	90	51	16.82	0.00E+00	30	11.185853	0.00E+00
GO:0009790	embryonic development	655	123	2.67	0.00E+00	104	2.76983	0.00E+00
GO:0021953	central nervous system neuron differentiation	93	58	13.04	0.00E+00	27	9.396117	0.00E+00
GO:0048646	anatomical structure formation	518	103	3.31	0.00E+00	83	3.282313	0.00E+00
GO:0051960	regulation of nervous system development	345	110	5.61	0.00E+00	65	4.712558	0.00E+00
GO:0022037	metencephalon development	64	39	17.53	0.00E+00	24	12.528155	0.00E+00
GO:0007420	brain development	409	213	11.09	0.00E+00	166	11.709875	0.00E+00
GO:0030902	hindbrain development	94	54	12.72	0.00E+00	36	12.528155	0.00E+00
GO:0000904	cellular morphogenesis during differentiation	478	154	5.33	0.00E+00	77	3.19004	0.00E+00
GO:0032989	cellular structure morphogenesis	634	175	4.39	0.00E+00	95	2.817649	0.00E+00
GO:0003002	regionalization	215	62	4.65	0.00E+00	52	5.121573	0.00E+00
GO:0048598	embryonic morphogenesis	345	84	3.78	0.00E+00	63	3.57461	0.00E+00
GO:0007409	axonogenesis	387	127	5.66	0.00E+00	69	3.915049	0.00E+00
GO:0050767	regulation of neurogenesis	313	101	5.68	0.00E+00	55	4.222111	0.00E+00
GO:0021549	cerebellum development	56	33	16.32	0.00E+00	21	15.660194	0.00E+00
GO:0048667	neuron morphogenesis during differentiation	410	137	5.55	0.00E+00	70	3.690955	0.00E+00
GO:0048666	neuron development	556	173	5.31	0.00E+00	89	3.182094	0.00E+00
GO:0045595	regulation of cell differentiation	765	149	3.36	0.00E+00	104	2.728074	0.00E+00
GO:0021915	neural tube development	94	38	8.95	0.00E+00	30	6.80878	4.44E-13
GO:0045664	regulation of neuron differentiation	261	86	5.91	0.00E+00	44	4.253386	3.11E-12
GO:0021987	cerebral cortex development	62	35	17.31	0.00E+00	19	11.020137	1.47E-11
GO:0035295	tube development	326	71	3.38	0.00E+00	54	3.396187	2.84E-11
GO:0021766	hippocampus development	44	25	20.61	0.00E+00	17	12.6773	2.98E-11
GO:0009887	organ morphogenesis	597	115	3.11	0.00E+00	81	2.578203	4.22E-11

GO:0022603	regulation of anatomical structure morphogenesis	440	94	3.34	0.00E+00	68	2.862616	4.62E-11
GO:0031399	regulation of protein modification process	831	109	2.2	5.77E-11	97	2.322689	5.95E-11
GO:0048585	negative regulation of response to stimulus	563	90	2.5	9.77E-12	75	2.645303	1.13E-10
GO:0009953	dorsal/ventral pattern formation	68	23	8.12	2.44E-11	23	7.503843	1.29E-10
GO:0009725	response to hormone stimulus	656	91	2.43	3.42E-11	76	2.592973	2.08E-10
GO:0051093	negative regulation of developmental process	401	80	3.6	0.00E+00	61	2.921321	4.24E-10
GO:0021536	diencephalon development	54	30	10.6	0.00E+00	21	7.830097	6.05E-10
GO:0006928	cell motility	701	117	2.72	0.00E+00	82	2.418335	8.41E-10
GO:0021872	generation of neurons in the forebrain	25	13	16.07	1.02E-09	12	20.880259	2.60E-10
GO:0021795	cerebral cortex cell migration	25	14	69.24	0.00E+00	8	41.760518	3.11E-09
GO:0016477	cell migration	483	89	3.14	0.00E+00	60	2.796463	4.07E-09
GO:0007267	cell-cell signaling	737	142	3.14	0.00E+00	84	2.307818	4.90E-09
GO:0051094	positive regulation of developmental process	535	97	3.12	0.00E+00	65	2.650814	5.09E-09
GO:0048854	brain morphogenesis	18	9	44.51	1.00E-10	12	15.660194	9.20E-09
GO:0051129	negative regulation of cellular component organization and biogenesis	256	49	3.15	7.50E-09	40	3.728618	3.82E-09
GO:0048568	embryonic organ development	241	53	2.95	1.25E-08	52	4.308625	0.00E+00
GO:0008283	cell proliferation	480	74	2.77	1.87E-11	61	2.675831	1.64E-08
GO:0009952	anterior/posterior pattern formation	150	36	3.87	1.66E-08	41	6.294784	0.00E+00
GO:0045165	cell fate commitment	156	43	3.94	1.01E-10	31	4.373568	1.91E-08
GO:0008284	positive regulation of cell proliferation	529	85	2.82	0.00E+00	63	2.610032	2.11E-08
GO:0031327	negative regulation of cellular biosynthetic process	758	131	2.99	0.00E+00	86	2.200616	3.09E-08
GO:0040007	growth	267	49	3.07	1.85E-08	41	3.508568	1.39E-08
GO:0016265	death	803	104	2.09	5.08E-09	90	2.155073	3.03E-08
GO:0008219	cell death	800	103	2.08	9.04E-09	90	2.155073	3.03E-08
GO:0030111	regulation of Wnt receptor signaling pathway	127	31	4.51	9.08E-09	25	5.220065	3.84E-08
GO:0007610	behavior	356	86	4.21	0.00E+00	49	2.974223	5.08E-08
GO:0001932	regulation of protein amino acid phosphorylation	678	91	2.26	2.03E-09	77	2.283778	6.83E-08
GO:0048870	cell motility involved in cell locomotion	520	93	2.99	0.00E+00	62	2.548378	8.12E-08
GO:0051674	localization of cell	520	93	2.99	0.00E+00	62	2.548378	8.12E-08
GO:0045596	negative regulation of cell differentiation	312	66	3.67	0.00E+00	49	2.906627	1.12E-07
GO:0021954	central nervous system neuron development	42	26	12.86	0.00E+00	10	17.400216	1.27E-07

GO:0021695	cerebellar cortex development	30	19	23.49	0.00E+00	12	12.528155	1.41E-07
GO:0021575	hindbrain morphogenesis	28	19	23.49	0.00E+00	11	14.355178	1.59E-07
GO:0009968	negative regulation of signal transduction	459	72	2.41	3.34E-08	59	2.588099	1.32E-07
GO:0009890	negative regulation of biosynthetic process	771	131	2.94	0.00E+00	86	2.127609	1.67E-07
GO:0031344	regulation of cell projection organization and biogenesis	202	54	5.68	0.00E+00	32	3.884699	1.92E-07
GO:0035270	endocrine system development	115	35	4.33	1.40E-09	23	5.220065	2.23E-07
GO:0043069	negative regulation of programmed cell death	505	85	3.14	0.00E+00	60	2.525838	2.36E-07
GO:0043066	negative regulation of apoptosis	500	84	3.12	0.00E+00	60	2.525838	2.36E-07
GO:0051130	positive regulation of cellular component organization and biogenesis	370	67	3.28	0.00E+00	52	2.741852	2.38E-07
GO:0048562	embryonic organ morphogenesis	155	38	3.36	2.89E-07	40	5.643313	0.00E+00
GO:0045934	negative regulation of nucleobase, nucleoside, nucleotide and nucleic acid metabolic process	700	123	3.01	0.00E+00	78	2.189059	3.64E-07
GO:0051253	negative regulation of RNA metabolic process	611	107	3.02	0.00E+00	71	2.287806	3.78E-07
GO:0045597	positive regulation of cell differentiation	401	76	3.45	0.00E+00	50	2.747402	5.00E-07
GO:0042493	response to drug	342	49	2.79	5.03E-07	50	3.182966	2.83E-09
GO:0048839	inner ear development	116	32	4.4	8.26E-09	28	4.176052	5.05E-07
GO:0001654	eye development	211	49	3.85	4.88E-12	38	3.251844	6.75E-07
GO:0051172	negative regulation of nitrogen compound metabolic process	710	124	2.99	0.00E+00	78	2.154312	7.54E-07
GO:0045892	negative regulation of transcription, DNA-dependent	589	106	3.08	0.00E+00	69	2.279649	7.95E-07
GO:0042325	regulation of phosphorylation	740	101	2.32	2.04E-11	81	2.114126	8.21E-07
GO:0009991	response to extracellular stimulus	344	54	2.59	8.44E-07	52	3.270402	3.66E-10
GO:0016055	Wnt receptor signaling pathway	139	30	3.8	1.19E-06	33	4.306553	6.59E-09
GO:0045944	positive regulation of transcription from RNA polymerase II promoter	503	107	3.31	0.00E+00	59	2.425069	1.57E-06
GO:0019220	regulation of phosphate metabolic process	792	109	2.29	4.00E-12	86	2.031337	1.59E-06
GO:0051174	regulation of phosphorus metabolic process	792	109	2.29	4.00E-12	86	2.031337	1.59E-06
GO:0043583	ear development	132	38	4.7	1.20E-11	29	3.784547	2.45E-06
GO:0016331	morphogenesis of embryonic epithelium	87	35	8.24	0.00E+00	23	4.61775	2.48E-06
GO:0021587	cerebellum morphogenesis	26	18	29.67	0.00E+00	10	13.050162	2.53E-06

GO:0035148	lumen formation	77	30	7.81	0.00E+00	21	4.982789	3.03E-06
GO:0040008	regulation of growth	421	63	2.29	4.15E-06	58	3.186987	4.53E-11
GO:0051050	positive regulation of transport	370	57	2.68	7.51E-08	48	2.637506	4.21E-06
GO:0048729	tissue morphogenesis	326	68	3.2	4.44E-13	47	2.638097	6.12E-06
GO:0021772	olfactory bulb development	20	12	14.84	1.80E-08	6	31.320388	6.22E-06
GO:0021988	olfactory lobe development	20	12	14.84	1.80E-08	6	31.320388	6.22E-06
GO:0007411	axon guidance	300	95	5.22	0.00E+00	42	2.810804	6.66E-06
GO:0060070	Wnt receptor signaling pathway through beta-catenin	64	18	5.56	6.70E-06	23	7.062441	4.61E-10
GO:0033273	response to vitamin	148	30	3.53	6.70E-06	27	4.698058	7.38E-08
GO:0008285	negative regulation of cell proliferation	433	74	2.67	1.18E-10	48	2.583125	8.17E-06
GO:0007584	response to nutrient	221	38	2.94	1.16E-05	34	3.549644	5.70E-07
GO:0001505	regulation of neurotransmitter levels	89	20	4.95	8.12E-06	21	4.766146	6.73E-06
GO:0048521	negative regulation of behavior	20	6	29.67	9.02E-06	6	31.320388	6.22E-06
GO:0031346	positive regulation of cell projection organization and biogenesis	105	23	4.95	6.63E-07	18	5.220065	1.82E-05
GO:0022029	telencephalon cell migration	32	21	25.96	0.00E+00	10	10.440129	2.47E-05
GO:0012501	programmed cell death	677	83	1.95	2.49E-05	76	2.179807	7.59E-07
GO:0007268	synaptic transmission	467	112	4.47	0.00E+00	53	2.364645	2.61E-05
GO:0030901	midbrain development	22	11	9.07	2.64E-05	13	13.572168	9.44E-09
GO:0043549	regulation of kinase activity	543	74	2.38	3.07E-08	60	2.221304	2.85E-05
GO:0019226	transmission of nerve impulse	527	131	4.8	0.00E+00	59	2.231767	3.19E-05
GO:0051338	regulation of transferase activity	554	75	2.36	2.93E-08	61	2.196027	3.27E-05
GO:0001838	embryonic epithelial tube formation	71	29	8.44	0.00E+00	19	4.722916	3.92E-05
GO:0021846	cell proliferation in forebrain	19	12	59.35	0.00E+00	8	13.920173	4.35E-05
GO:0001841	neural tube formation	59	24	9.13	4.44E-13	17	5.220065	4.40E-05
GO:0031667	response to nutrient levels	321	48	2.45	4.60E-05	50	3.676102	1.29E-11
GO:0021879	forebrain neuron differentiation	17	10	12.36	4.43E-06	8	13.920173	4.35E-05
GO:0045859	regulation of protein kinase activity	518	70	2.32	2.90E-07	57	2.237171	5.13E-05
GO:0035239	tube morphogenesis	211	51	3.71	5.33E-12	34	2.958037	5.60E-05
GO:0001944	vasculature development	343	51	2.36	5.67E-05	46	3.039531	9.93E-08
GO:0048589	developmental growth	159	36	3.96	8.90E-09	25	3.625045	7.05E-05
GO:0021904	dorsoventral neural tube patterning	15	8	13.19	6.90E-05	6	31.320388	6.22E-06
GO:0048545	response to steroid hormone stimulus	324	49	2.72	1.07E-06	39	2.678717	8.47E-05
GO:0014020	primary neural tube	50	21	8.65	8.66E-11	14	6.090076	8.73E-05

	formation							
GO:0042471	ear morphogenesis	74	20	4.3	8.69E-05	22	5.220065	5.39E-07
GO:0050769	positive regulation of neurogenesis	102	30	4.79	4.40E-09	18	4.698058	9.47E-05
GO:0009798	axis specification	64	18	4.69	1.00E-04	18	5.872573	2.77E-06
GO:0001764	neuron migration	77	30	7.81	0.00E+00	16	4.395844	1.16E-03
GO:0048863	stem cell differentiation	77	32	6.88	0.00E+00	15	4.605939	1.34E-03
GO:0016192	vesicle-mediated transport	680	85	2.03	2.28E-06	75	1.80417	2.17E-03
GO:0050770	regulation of axonogenesis	84	28	6.29	2.93E-11	15	4.350054	2.80E-03
GO:0007167	enzyme linked receptor protein signaling pathway	583	85	2.3	5.29E-09	56	1.988596	2.98E-03
GO:0001843	neural tube closure	48	21	8.65	8.66E-11	12	5.220065	3.64E-03
GO:0031345	negative regulation of cell projection organization and biogenesis	63	20	6.18	1.59E-07	12	5.220065	3.64E-03
GO:0051969	regulation of transmission of nerve impulse	176	53	7.28	0.00E+00	24	2.982894	4.45E-03
GO:0022604	regulation of cell morphogenesis	222	58	4.16	0.00E+00	33	2.460888	5.58E-03
GO:0043523	regulation of neuron apoptosis	138	34	4.54	7.43E-10	22	3.103822	5.82E-03
GO:0045665	negative regulation of neuron differentiation	42	14	6.92	1.66E-05	10	5.800072	7.99E-03
GO:0007611	learning and/or memory	141	41	6.54	0.00E+00	21	3.132039	8.20E-03
GO:0021515	cell differentiation in spinal cord	31	16	9.89	7.81E-09	8	6.960086	1.34E-02
GO:0050804	regulation of synaptic transmission	162	48	7.42	0.00E+00	22	2.944652	1.37E-02
GO:0006836	neurotransmitter transport	91	20	4.3	8.69E-05	18	3.355756	1.38E-02
GO:0021696	cerebellar cortex morphogenesis	19	14	69.24	0.00E+00	6	7.830097	6.21E-02
GO:0021895	cerebral cortex neuron differentiation	12	9	44.51	1.00E-10	5	8.700108	1.21E-01
GO:0021680	cerebellar Purkinje cell layer development	17	10	49.46	1.78E-12	5	8.700108	1.21E-01
GO:0021955	central nervous system neuron axonogenesis	21	14	17.31	5.42E-11	4	20.880259	1.00E+00
GO:0046907	intracellular transport	827	79	1.58	1.36E-01	111	2.41428	0.00E+00
GO:0021854	hypothalamus development	14	5	6.18	7.73E-01	9	46.980583	5.86E-11
GO:0046903	secretion	375	48	2.01	1.26E-02	55	2.84261	1.81E-08
GO:0035282	segmentation	56	15	3.9	1.11E-02	18	6.264078	9.72E-07
GO:0010243	response to organic nitrogen	134	25	3.02	2.35E-03	24	4.640058	1.03E-06
GO:0042472	inner ear morphogenesis	64	16	3.96	4.85E-03	20	5.494805	1.27E-06
GO:0031099	regeneration	109	17	2.9	1.60E-01	21	4.982789	3.03E-06
GO:0032107	regulation of response to nutrient levels	23	0	0	1.00E+00	6	31.320388	6.22E-06
GO:0032104	regulation of response to extracellular stimulus	23	0	0	1.00E+00	6	31.320388	6.22E-06
GO:0017145	stem cell division	17	6	7.42	8.75E-02	6	31.320388	6.22E-06

GO:0016568	chromatin modification	336	41	1.73	1.00E+00	45	2.700033	6.48E-06
GO:0032940	secretion by cell	299	42	2.16	8.04E-03	43	2.771145	6.66E-06
GO:0003001	generation of a signal involved in cell-cell signaling	111	23	3.07	4.28E-03	21	4.766146	6.73E-06
GO:0031329	regulation of cellular catabolic process	409	57	1.89	1.15E-02	51	2.511541	6.92E-06
GO:0051169	nuclear transport	237	20	1.71	1.00E+00	36	3.080694	7.93E-06
GO:0006913	nucleocytoplasmic transport	235	20	1.77	1.00E+00	36	3.080694	7.93E-06
GO:0048704	embryonic skeletal morphogenesis	57	15	3.09	1.85E-01	14	7.308091	8.05E-06
GO:0051090	regulation of transcription factor activity	254	32	1.72	1.00E+00	39	2.908322	9.27E-06
GO:0051247	positive regulation of protein metabolic process	469	51	1.84	7.22E-02	53	2.405769	1.47E-05
GO:0051276	chromosome organization and biogenesis	528	53	1.6	1.00E+00	61	2.242422	1.53E-05
GO:0033043	regulation of organelle organization and biogenesis	357	46	2.01	1.91E-02	52	2.423601	1.60E-05
GO:0000278	mitotic cell cycle	507	40	1.39	1.00E+00	60	2.253265	1.71E-05
GO:0045787	positive regulation of cell cycle	94	12	1.98	1.00E+00	19	4.959061	1.77E-05
GO:0000086	G2/M transition of mitotic cell cycle	112	11	2.09	1.00E+00	22	4.253386	2.46E-05
GO:0032270	positive regulation of cellular protein metabolic process	413	48	1.99	1.57E-02	49	2.459454	2.69E-05
GO:0010035	response to inorganic substance	336	43	2.31	1.12E-03	44	2.580706	3.63E-05
GO:0045740	positive regulation of DNA replication	37	6	2.7	1.00E+00	9	11.745146	3.69E-05
GO:0006886	intracellular protein transport	513	51	1.74	3.02E-01	62	2.157627	4.73E-05
GO:0001558	regulation of cell growth	242	33	2.12	1.21E-01	33	3.022143	5.30E-05
GO:0051329	interphase of mitotic cell cycle	293	23	1.5	1.00E+00	38	2.755034	6.03E-05
GO:0001701	in utero embryonic development	259	35	1.94	4.10E-01	37	2.799165	6.03E-05
GO:0051704	multi-organism process	830	78	1.58	1.55E-01	85	1.880108	6.77E-05
GO:0006325	establishment and/or maintenance of chromatin architecture	404	48	1.84	1.23E-01	48	2.409261	7.07E-05
GO:0051325	interphase	305	23	1.44	1.00E+00	38	2.717294	8.63E-05

Table S8 GO biological processes associated with proximal and *tele* lung enhancers

GOID	GO	#genes	Proximal			Tele		
			#genes	Enrichment Fold	p-value	#genes	Enrichment Fold	p-value
GO:0030323	respiratory tube development	127	63	14.47	0.00E+00	54	14.351829	0.00E+00
GO:0009725	response to hormone stimulus	656	94	2.97	0.00E+00	81	3.146362	0.00E+00
GO:0035295	tube development	326	90	6.02	0.00E+00	69	4.907472	0.00E+00
GO:0009719	response to endogenous stimulus	805	116	2.85	0.00E+00	94	2.912107	0.00E+00
GO:0009611	response to wounding	817	119	2.83	0.00E+00	102	2.488267	0.00E+00
GO:0030324	lung development	123	63	15.12	0.00E+00	52	14.588073	0.00E+00
GO:0009967	positive regulation of signal transduction	603	103	3.26	0.00E+00	78	2.773788	2.66E-12
GO:0008284	positive regulation of cell proliferation	529	89	3.46	0.00E+00	69	2.927987	9.33E-12
GO:0043068	positive regulation of programmed cell death	504	67	2.97	1.20E-11	71	2.86824	1.02E-11
GO:0043065	positive regulation of apoptosis	500	67	2.97	1.20E-11	71	2.86824	1.02E-11
GO:0014070	response to organic cyclic substance	222	35	3.24	3.42E-06	25	2.744412	1.24E-02
GO:0048598	embryonic morphogenesis	345	62	3.48	0.00E+00	39	2.188211	1.28E-02
GO:0045596	negative regulation of cell differentiation	312	69	4.34	0.00E+00	34	2.32014	1.42E-02
GO:0035239	tube morphogenesis	211	46	4.34	4.44E-13	26	2.431345	7.02E-02
GO:0030855	epithelial cell differentiation	180	40	3.99	4.69E-10	28	2.524859	1.82E-02
GO:0008283	cell proliferation	480	69	2.6	2.41E-09	48	1.970621	2.06E-02
GO:0030029	actin filament-based process	274	51	3.64	1.02E-11	32	2.341898	2.14E-02
GO:0033157	regulation of intracellular protein transport	116	23	4.5	4.09E-06	16	3.512847	2.26E-02
GO:0012502	induction of programmed cell death	322	43	3.11	1.99E-07	38	2.156059	2.34E-02
GO:0055093	response to hyperoxia	21	5	3.77	1.00E+00	10	16.832392	1.79E-07
GO:0007585	respiratory gaseous exchange	36	5	2.2	1.00E+00	12	12.119322	2.09E-07
GO:0055082	cellular chemical homeostasis	448	45	1.92	7.66E-02	51	2.627914	1.48E-06
GO:0046039	GTP metabolic process	213	19	1.45	1.00E+00	38	3.094988	2.62E-06
GO:0006184	GTP catabolic process	205	18	1.44	1.00E+00	37	3.113992	3.61E-06
GO:0045730	respiratory burst	15	0	0	1.00E+00	6	30.298305	7.80E-06

Table S9 Nucleotide divergence (per kilobase) of *tele* and proximal enhancers in cell types.

cell	proximal			tele			p-value (tele vs proximal)
	Non-human	human	NI	Non-human	human	NI	
GM12878	59.1112	5.3352	0.74	59.7612	5.2197	0.71	5.98E-06
H1-Hesc	56.4927	5.5512	0.8	58.2957	5.3274	0.75	6.03E-08
HepG2	58.1545	5.4365	0.76	59.4398	5.3354	0.73	6.83E-07
HSMM	54.7468	5.2121	0.78	56.553	5.254	0.76	2.13E-03
HUVEC	55.904	5.3227	0.78	57.6571	5.2051	0.74	2.36E-11
K562	58.6319	5.4612	0.76	60.7164	5.4047	0.73	6.98E-08
NHEK	56.6584	5.2833	0.76	58.1341	5.1209	0.72	1.97E-15
