# Supplementary material for: Genome-Wide Analysis of Functional and Evolutionary Features of Tele-Enhancers
Source: G3 (Bethesda). 2014 Feb 4;4(4):579–93. doi: 10.1534/g3.114.010447 (PMC4059231; doi:10.1534/g3.114.010447)
Supplement: Supporting Information [file supp_g3.114.010447_FileS1.pdf]

**File S1**

**Heart developmental genes**

We compiled heart developmental genes from two sources – gene expression data and gene annotation. Using geome-wide gene expression profiles across 79 tissues/cells (Su, Wiltshire et al. 2004, see URLs) we ranked genes according to their absolute expression in heart (denated as AbsoluteRank), and to their relative expression in heart (denoted as RelativeRank). We evaluated top 20% genes in these gene lists using genes annotated to heart development and heart disease. Using the genes fallen into the category of heart development (GO:0007507) and its children categories, we collected 348 genes, and noticed that top 20% RelativeRank genes are most enriched for these heart genes (Figure S1). Similarly, we obtained a list of 71 heart-disease-related genes reported in GeneTests (see URLs), and observed that top 20% RelativeRank display highest enrichment for these genes (Figure S1). Finally we checked the distribution of the genes in the neighborhood of p300-bound heart enhancers (i.e., the enhancers used in this study) along ranked gene lists. More specifically, we collected all genes of which TSSs are in a distance of less-than-10k-bp to any p300-bound heart enhancers, Theinvestigation of distribution of these heart-enhancer-nearby genes show that these genes are enriched in top 20% RelativeRank. These evaluation results indicated that the top 20% RelativeRank gene list show the most association with heart development/disease, and thus were adopted in this study. After combining top 20% RelativeRank genes with the genes fallen into heart development category in GO, we finally obtained 2430 distinct heart developmental genes for further investigation.
