# Supplementary material for: Genome-Wide Analysis of Functional and Evolutionary Features of Tele-Enhancers
Source: G3 (Bethesda). 2014 Feb 4;4(4):579–93. doi: 10.1534/g3.114.010447 (PMC4059231; doi:10.1534/g3.114.010447)
Supplement: Supporting Information [file supp_g3.114.010447_FigureS1.pdf]

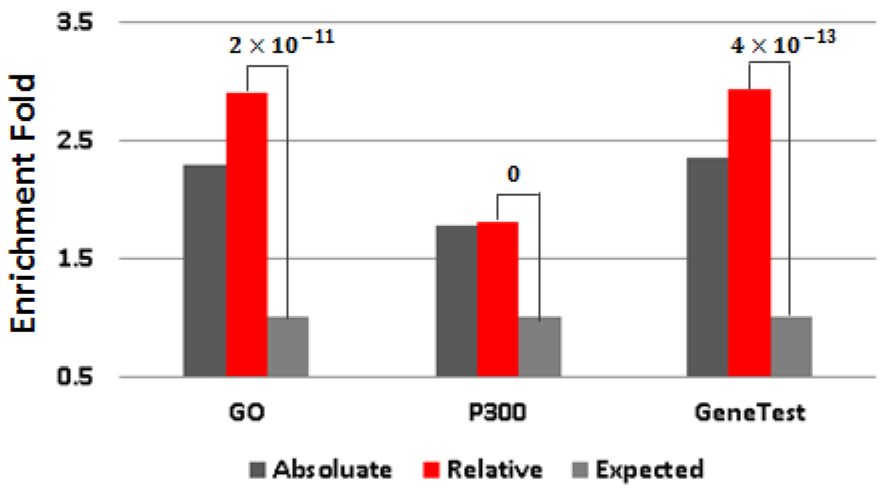

Figure S1 Comparisons between AbsoluteRank and RelativeRank based on the enrichment of GO heart development genes, nearby genes of p300-bound heart enhancers and GeneTest heart disease genes.
