# Supplementary material for: Genome-Wide Analysis of Functional and Evolutionary Features of Tele-Enhancers
Source: G3 (Bethesda). 2014 Feb 4;4(4):579–93. doi: 10.1534/g3.114.010447 (PMC4059231; doi:10.1534/g3.114.010447)
Supplement: Supporting Information [file supp_g3.114.010447_FigureS2.pdf]

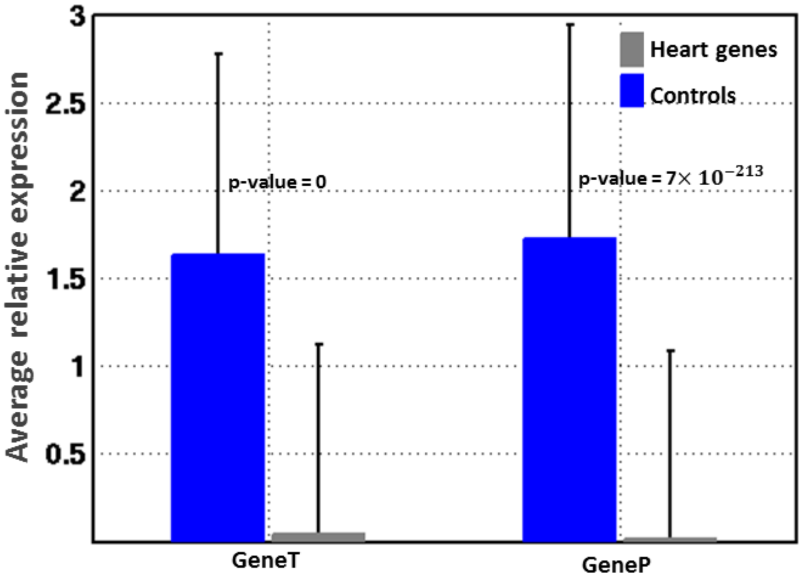

Figure S2 Comparisons of relative expression between heart genes and controls. Heart genes are grouped into GeneTs, which are associated with *tele*-enhancers, and GenePs, associated with proximal enhancers.
