# Supplementary material for: Genome-Wide Analysis of Functional and Evolutionary Features of Tele-Enhancers
Source: G3 (Bethesda). 2014 Feb 4;4(4):579–93. doi: 10.1534/g3.114.010447 (PMC4059231; doi:10.1534/g3.114.010447)
Supplement: Supporting Information [file supp_g3.114.010447_FigureS3.pdf]

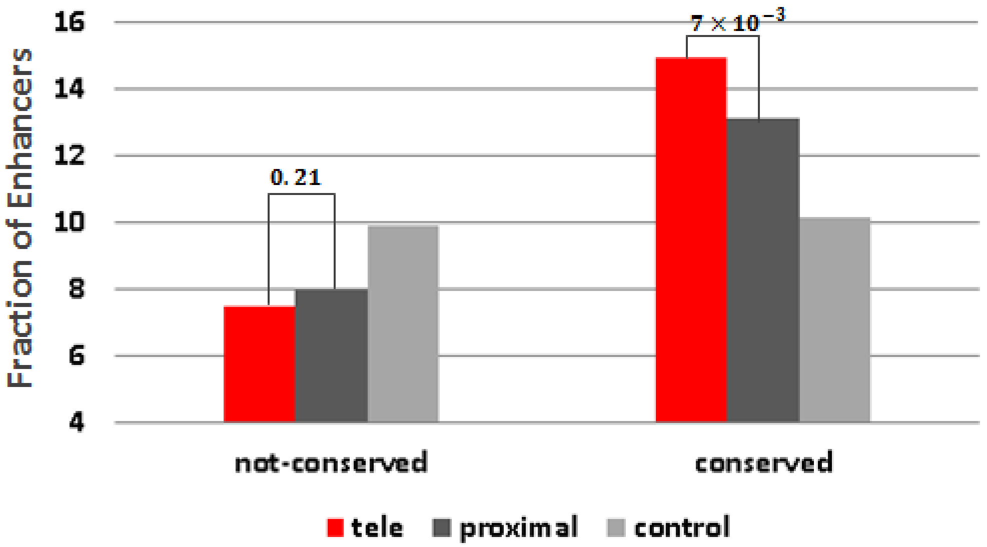

Figure S3 Fraction of not-conserved and conserved enhancers in *tele* and proximal heart enhancers. The conservation levels of sequences (either enhancers or controls) were estimated as the average of phastcon score. A sequence having an average phastcon score of < 0.05 was regarded as not-conserved, while an average phastcon score of >0.2 indicates a conserved sequence. The cutoff values for not-conserved and conserved were determined with reference to controls that are randomly-selected non-coding sequences having similar GC content, repeat density and same length to enhancers. After controls based on the conservation levels, we set the cutoff values so that 10% controls are conserved, and 10% controls are not conserved.
