# Supplementary material for: Genome-Wide Analysis of Functional and Evolutionary Features of Tele-Enhancers
Source: G3 (Bethesda). 2014 Feb 4;4(4):579–93. doi: 10.1534/g3.114.010447 (PMC4059231; doi:10.1534/g3.114.010447)
Supplement: Supporting Information [file supp_g3.114.010447_FigureS4.pdf]

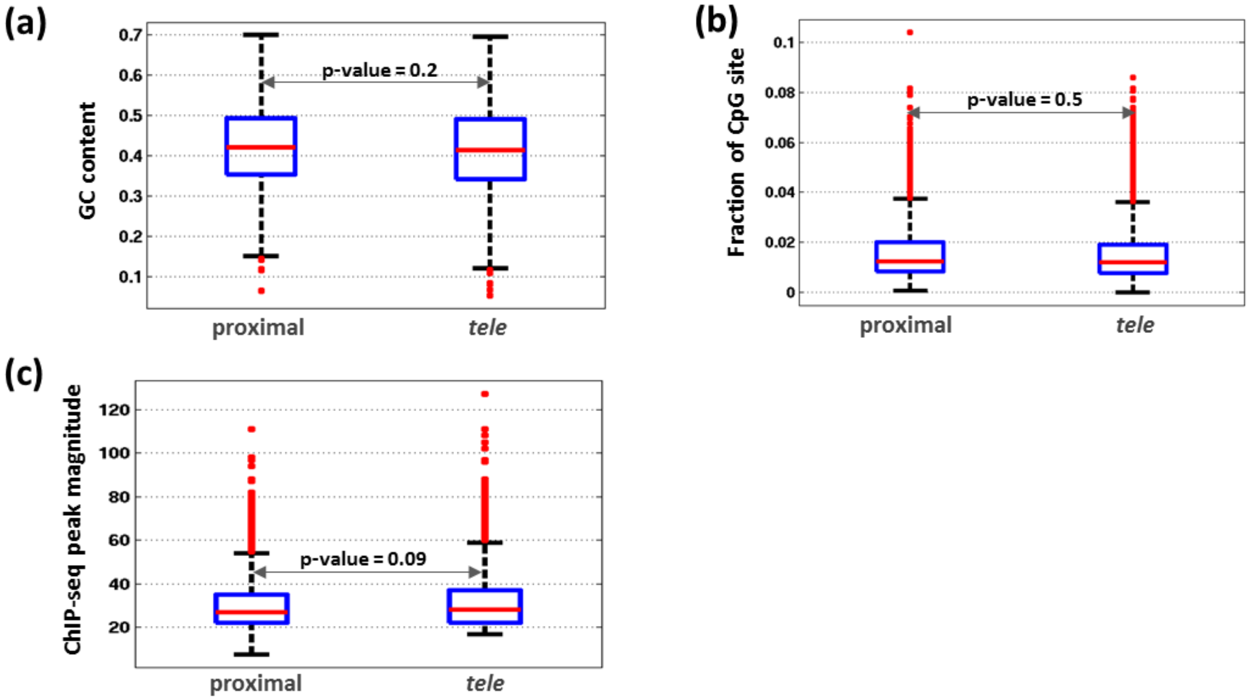

Figure S4 Comparison of tele and proximal heart enhancers in terms of GC content, CpC site density and p300 ChIP-seq peak signal.
