# Supplementary material for: Genome-Wide Analysis of Functional and Evolutionary Features of Tele-Enhancers
Source: G3 (Bethesda). 2014 Feb 4;4(4):579–93. doi: 10.1534/g3.114.010447 (PMC4059231; doi:10.1534/g3.114.010447)
Supplement: Supporting Information [file supp_g3.114.010447_TableS1.pdf]

Table S1 Distribution of GeneTs, GenePs, *tele* and proximal enhancers in fetal brain and lung.

	# Genes			# Enhancers			Fraction of multiple-tissue genes (%)			
	All	GeneT	GeneP	All	enT	enP	GeneA	GeneT	GeneP	pvalue (GeneT v.s. GeneP)
Brain	2689	927	1030	9604	4273	3376	28	30.1	26.3	0.12
Lung	2434	864	852	8934	3153	2816	58.3	60.3	55.8	0.09
