# Supplementary material for: Genome-Wide Analysis of Functional and Evolutionary Features of Tele-Enhancers
Source: G3 (Bethesda). 2014 Feb 4;4(4):579–93. doi: 10.1534/g3.114.010447 (PMC4059231; doi:10.1534/g3.114.010447)
Supplement: Supporting Information [file supp_g3.114.010447_TableS2.pdf]

Table S2 Distribution of GeneTs, GenePs, *tele* and proximal enhancers in cell types.

	#Gene	#GeneP	#GeneT	#enhancer	#proximal Enhancer	#tel-enhancer
GM12878	2398	1801	575	64090	21890	5730
H1-Hesc	2398	1250	1095	18076	4853	3332
HepG2	2398	1802	555	45107	17249	4261
HSMM	2398	1990	391	81275	30271	3642
HUVEC	2398	2068	312	79127	27915	3731
K562	2398	2022	344	69096	18351	4735
NHEK	2398	1996	388	86529	26526	5526
