# Supplementary material for: Genome-Wide Analysis of Functional and Evolutionary Features of Tele-Enhancers
Source: G3 (Bethesda). 2014 Feb 4;4(4):579–93. doi: 10.1534/g3.114.010447 (PMC4059231; doi:10.1534/g3.114.010447)
Supplement: Supporting Information [file supp_g3.114.010447_TableS3.pdf]

Table S3 GO biological processes associated with *tele* and proximal heart enhancers.

GOID	GO	Number of Genes	Proximal (associated with 661 genes)			Tele (associated with 1171 genes)		
			Number of Genes	Enrichment Fold	p-value	Number of Genes	Enrichment Fold	p-value
GO:0007507	heart development	297	120	13.13	0.00E+00	131	12.812562	0.00E+00
GO:0003007	heart morphogenesis	131	56	17.67	0.00E+00	55	11.46029	0.00E+00
GO:0035050	embryonic heart tube development	42	18	19.31	0.00E+00	20	15.97495	0.00E+00
GO:0048738	cardiac muscle development	85	45	24.13	0.00E+00	35	11.981213	0.00E+00
GO:0001944	vasculature development	343	89	7.96	0.00E+00	58	4.087708	0.00E+00
GO:0003012	muscle system process	185	42	7.77	0.00E+00	46	5.801429	0.00E+00
GO:0048729	tissue morphogenesis	326	72	5.29	0.00E+00	80	5.044721	0.00E+00
GO:0007517	muscle development	205	49	6.92	0.00E+00	48	4.694679	0.00E+00
GO:0009790	embryonic development	655	108	4.35	0.00E+00	112	2.710901	0.00E+00
GO:0035295	tube development	326	80	7.03	0.00E+00	70	3.727488	0.00E+00
GO:0001568	blood vessel development	322	84	8.5	0.00E+00	56	4.399658	0.00E+00
GO:0006936	muscle contraction	160	38	7.84	0.00E+00	43	6.061084	0.00E+00
GO:0014706	striated muscle development	178	57	9.26	0.00E+00	49	6.179783	0.00E+00
GO:0035239	tube morphogenesis	211	54	6.74	0.00E+00	47	4.416604	0.00E+00
GO:0001701	in utero embryonic development	259	43	4.8	0.00E+00	55	4.118542	0.00E+00
GO:0002009	morphogenesis of an epithelium	242	57	5.36	0.00E+00	49	4.048824	8.88E-13
GO:0007389	pattern specification process	301	54	3.86	0.00E+00	57	3.502201	1.78E-12
GO:0055010	ventricular cardiac muscle morphogenesis	28	10	53.63	8.88E-13	15	17.971819	4.44E-12
GO:0035051	cardiac cell differentiation	62	34	36.47	0.00E+00	20	10.649967	6.22E-12
GO:0048598	embryonic morphogenesis	345	69	5.44	0.00E+00	65	3.054035	1.15E-11
GO:0001947	heart looping	32	14	18.77	1.60E-11	16	15.335952	7.99E-12
GO:0042692	muscle cell differentiation	184	56	8.12	0.00E+00	38	4.441815	7.37E-11
GO:0048514	blood vessel morphogenesis	271	72	8.78	0.00E+00	43	3.816238	3.10E-10
GO:0055008	cardiac muscle morphogensis	36	13	17.43	3.34E-10	20	19.16994	0.00E+00
GO:0048644	muscle morphogenesis	45	15	11.49	3.41E-09	20	13.692814	0.00E+00
GO:0055007	cardiac muscle cell differentiation	45	29	38.88	0.00E+00	15	10.269611	1.91E-08
GO:0055001	muscle cell development	82	33	16.09	0.00E+00	21	5.920129	1.32E-07
GO:0001501	skeletal development	304	56	4.48	0.00E+00	46	2.755679	2.57E-06
GO:0051146	striated muscle cell differentiation	122	45	12.7	0.00E+00	25	4.279005	2.64E-06
GO:0055006	cardiac cell development	26	16	85.81	0.00E+00	9	14.377455	5.58E-06
GO:0055013	cardiac muscle cell development	26	16	85.81	0.00E+00	9	14.377455	5.58E-06
GO:0001570	vasculogenesis	55	22	19.66	0.00E+00	15	6.535207	1.22E-05
GO:0055002	striated muscle cell development	63	25	16.76	0.00E+00	15	5.990606	4.06E-05

GO:0001569	patterning of blood vessels	26	12	8.04	2.67E-05	8	12.77996	9.03E-05
GO:0003013	circulatory system process	222	41	6.28	0.00E+00	32	2.323629	2.64E-02
GO:0008015	blood circulation	217	39	5.98	0.00E+00	32	2.323629	2.64E-02
GO:0003015	heart process	30	12	64.36	0.00E+00	8	6.38998	2.66E-02
GO:0060047	heart contraction	26	11	58.99	0.00E+00	8	6.38998	2.66E-02
GO:0048844	artery morphogenesis	27	13	69.72	0.00E+00	7	6.709479	5.44E-02
GO:0006937	regulation of muscle contraction	77	19	7.84	6.44E-09	16	3.06719	1.26E-01
GO:0043433	negative regulation of transcription factor activity	85	17	5.07	6.60E-05	16	3.06719	1.26E-01
GO:0048771	tissue remodeling	73	15	8.04	6.15E-07	12	3.594364	1.84E-01
GO:0001974	blood vessel remodeling	27	9	48.27	4.44E-11	6	5.750982	4.26E-01
GO:0060070	Wnt receptor signaling pathway through beta-catenin	64	16	7.8	2.79E-07	12	3.382931	3.34E-01
GO:0016202	regulation of striated muscle development	73	22	10.73	4.44E-13	12	3.026833	9.75E-01
GO:0043535	regulation of blood vessel endothelial cell migration	30	11	9.83	1.06E-05	5	3.993738	1.00E+00
GO:0060039	pericardium development	12	8	14.3	3.41E-05	4	6.38998	1.00E+00
GO:0048010	vascular endothelial growth factor receptor signaling pathway	22	6	32.18	5.13E-06	5	4.792485	1.00E+00
GO:0048660	regulation of smooth muscle cell proliferation	64	13	7.75	1.22E-05	8	2.255287	1.00E+00
GO:0043552	positive regulation of phosphoinositide 3-kinase activity	24	6	32.18	5.13E-06	0	0	1.00E+00
GO:0031668	cellular response to extracellular stimulus	150	26	7.75	1.33E-12	19	1.979505	1.00E+00
GO:0043269	regulation of ion transport	278	35	3.35	1.26E-06	32	1.870238	1.00E+00
GO:0006942	regulation of striated muscle contraction	30	10	17.88	9.35E-08	6	3.19499	1.00E+00
GO:0055024	regulation of cardiac muscle development	27	12	64.36	0.00E+00	6	4.792485	1.00E+00
GO:0055012	ventricular cardiac muscle cell differentiation	18	11	58.99	0.00E+00	3	2.875491	1.00E+00
GO:0055015	ventricular cardiac muscle cell development	13	7	37.54	1.18E-07	3	4.792485	1.00E+00
GO:0048705	skeletal morphogenesis	142	30	7	0.00E+00	18	2.331479	1.00E+00
GO:0045333	cellular respiration	126	11	2.46	1.00E+00	40	7.099978	0.00E+00
GO:0015980	energy derivation by oxidation of organic compounds	258	22	2.11	1.00E+00	56	4.969985	0.00E+00
GO:0052548	regulation of endopeptidase activity	240	17	1.6	1.00E+00	47	3.633013	1.45E-10
GO:0052547	regulation of peptidase activity	252	18	1.69	1.00E+00	48	3.539066	2.07E-10
GO:0002697	regulation of immune effector process	165	13	2.49	1.00E+00	36	4.313237	7.77E-10
GO:0007005	mitochondrion organization	149	14	2.35	1.00E+00	36	4.208036	1.59E-09

	and biogenesis							
GO:0031099	regeneration	109	9	1.61	1.00E+00	26	5.663846	2.68E-09
GO:0006818	hydrogen transport	97	8	2.52	1.00E+00	26	5.191859	1.90E-08
GO:0015992	proton transport	96	8	2.52	1.00E+00	26	5.191859	1.90E-08
GO:0006979	response to oxidative stress	203	16	2.32	1.00E+00	38	3.642289	2.88E-08
GO:0006119	oxidative phosphorylation	47	4	2.68	1.00E+00	18	7.188728	1.03E-07
GO:0032374	regulation of cholesterol transport	30	0	0	1.00E+00	7	33.547395	2.90E-07
GO:0032371	regulation of sterol transport	30	0	0	1.00E+00	7	33.547395	2.90E-07
GO:0006120	mitochondrial electron transport, NADH to ubiquinone	32	3	2.3	1.00E+00	14	8.386849	1.30E-06
GO:0042775	organelle ATP synthesis coupled electron transport	41	4	2.68	1.00E+00	15	7.188728	3.23E-06
GO:0042773	ATP synthesis coupled electron transport	41	4	2.68	1.00E+00	15	7.188728	3.23E-06
GO:0006839	mitochondrial transport	76	6	1.61	1.00E+00	20	5.044721	5.86E-06
GO:0051186	cofactor metabolic process	219	15	1.71	1.00E+00	38	2.845538	2.78E-05
GO:0043122	regulation of I-kappaB kinase/NF-kappaB cascade	140	9	1.34	1.00E+00	26	3.664842	3.25E-05
GO:0030811	regulation of nucleotide catabolic process	273	23	1.62	1.00E+00	38	2.80176	4.18E-05
GO:0033121	regulation of purine nucleotide catabolic process	273	23	1.62	1.00E+00	38	2.80176	4.18E-05
GO:0031334	positive regulation of protein complex assembly	71	7	1.5	1.00E+00	16	5.477126	5.47E-05
GO:0002673	regulation of acute inflammatory response	36	0	0	1.00E+00	10	9.58497	5.93E-05
GO:0002757	immune response-activating signal transduction	171	9	1.79	1.00E+00	30	3.19499	6.68E-05
GO:0051348	negative regulation of transferase activity	138	16	2	1.00E+00	25	3.63067	7.13E-05
GO:0009308	amine metabolic process	457	27	1.34	1.00E+00	63	2.126243	7.17E-05
