# Supplementary material for: Genome-Wide Analysis of Functional and Evolutionary Features of Tele-Enhancers
Source: G3 (Bethesda). 2014 Feb 4;4(4):579–93. doi: 10.1534/g3.114.010447 (PMC4059231; doi:10.1534/g3.114.010447)
Supplement: Supporting Information [file supp_g3.114.010447_TableS4.pdf]

Table S4 Weights of binding motifs in linear SVMs built for *tele* and proximal heart enhancers.

Rank	Motif	*Tele*	Proximal
1	RSRFC4 01	229	141
2	MEF2 02	85	174
3	MEF2A	101	98
4	MMEF2 Q6	110	82
5	GATA1 04	101	65
6	TST1 01	75	67
7	HMEF2 Q6	63	78
8	GAF Q6	62	63
9	NR4A2	99	60
10	SOX4 01	67	58
11	NF1 Q6	104	48
12	MEF2 05	126	20
13	SOX Q6	88	55
14	HOXB4 01	136	0
15	EFC Q6	112	21
16	FOXD1	87	45
17	DBX2 01	124	0
18	SMAD1 01	118	3
19	CTF1 01	87	32
20	GBX2 01	119	0
21	P53 05	84	32
22	SOX9	110	0
23	Gata1	66	39
24	ERR1 Q2	82	20
25	HNF1A	75	23
26	PXR Q2	62	35
27	SOX9 Q4	79	16
28	SRF 02	76	16
29	AP4 Q5	65	22
30	TCF11MAFG 01	85	0
31	RAX 01	77	6
32	CLOCKBMAL Q6	90	-9
33	BRCA 01	81	0
34	IRX5 01	110	-32
35	TFE Q6	76	0
36	BRN4 01	76	0
37	NKX21 01	72	0
38	NKX25 Q5	72	0
39	DOBOX5 01	69	0
40	HOXB5 01	68	0
41	DMRT1 01	71	-5
42	LMX1B 01	66	0
43	GATA Q6	65	0
44	SRF C	61	4
45	CREB 01	64	0
46	MTATA B	76	-23
47	HEB Q6	85	-38
48	HOXA10 01	88	-45
49	FOXP3 Q4	67	-30
50	Mafb	69	-37
51	NKX63 01	93	-71
52	CART1 02	65	-55
53	MSX3 01	106	-96
54	LHX3 01	145	-212
55	AP1 Q4 01	41	183
56	Sox2	0	180

57	STAT6 01	0	175
58	POU1F1 Q6	19	148
59	MEF2 Q6 01	48	115
60	NCX 02	44	117
61	AP1 Q6 01	-1	151
62	CEBP 01	0	142
63	FOXO4 01	0	138
64	HNF1B 01	26	109
65	NKX52 01	24	96
66	SRY	0	118
67	MRG2 01	0	110
68	STAT3 03	25	85
69	PITX3 Q2	0	105
70	LHX5 01	-62	165
71	HOXB7 01	40	62
72	HNF4 Q6 01	38	63
73	PAX1 B	0	99
74	MYB Q3	18	81
75	REL	0	96
76	EVI1 05	-21	116
77	MAX	0	95
78	FXR Q2	0	93
79	MEF2 04	-14	105
80	OTX Q1	21	67
81	HNF3A 01	16	69
82	BRCA1	0	84
83	MYOGNF1 01	6	78
84	HNF1 Q6	16	66
85	VSX1 01	-25	107
86	PITX3 01	0	81
87	SRF 01	0	80
88	DLX1 01	16	63
89	Nobox	0	79
90	GATA4 Q3	0	78
91	RSRFC4 Q2	16	62
92	IPF1 Q4	14	64
93	CIZ 01	0	77
94	NRF2 Q4	-3	80
95	HSF2 02	0	75
96	BEN 02	0	75
97	CREB Q2	14	61
98	NKX3A 02	10	63
99	DLX7 01	0	73
100	CDX1 01	0	71
101	FEV	0	71
102	FOXD3 01	0	69
103	USF C	-3	72
104	MTF1 01	0	67
105	IRF2 01	0	62
106	TITF1 Q3	0	61
107	LEF1TCF1 Q4	0	60
108	TR4 Q2	-9	62
109	SHOX2 01	-45	94
110	IPF1 Q4 01	-49	95
111	Lhx3	-22	66
112	LHX3 02	-119	161
113	CEBPGAMMA Q6	-87	130
114	SP1 Q4 01	-53	83

115	TP53	-57	75
116	HOXC10 01	-86	67
117	VDRRXR 01	-96	65
