# Supplementary material for: Genome-Wide Analysis of Functional and Evolutionary Features of Tele-Enhancers
Source: G3 (Bethesda). 2014 Feb 4;4(4):579–93. doi: 10.1534/g3.114.010447 (PMC4059231; doi:10.1534/g3.114.010447)
Supplement: Supporting Information [file supp_g3.114.010447_TableS5.pdf]

Table S5 Enrichment Fold of TF binding motifs along *tele* and proximal heart enhancer sequences.

Motif	*Tele*	Proximal
MEF2 02	2.33	2.04
MEF2 03	2.12	1.95
RSRFC4 01	2.17	1.95
MEF2A	2.67	1.86
RSRFC4 Q2	2.46	1.84
MEF2 05	2.6	1.71
HMEF2 Q6	2.62	1.6
MEF2 Q6 01	3.12	1.59
NR4A2	1.45	1.5
ESR2	1.44	1.4
PXR Q2	1.51	1.39
RORA2 01	1.38	1.4
AMEF2 Q6	1.89	1.35
ELK1 02	1.35	1.42
TR4 Q2	1.34	1.34
Esrrb	1.39	1.33
ERR2 01	1.32	1.33
AP2 Q6 01	1.32	1.42
NFKB Q6 01	1.31	1.42
NFKAPPAB 01	1.33	1.31
BEN 02	1.33	1.3
DBP Q6	1.4	1.3
PAX3 01	1.52	1.3
NF1 Q6	1.6	1.29
LRH1 Q5	1.33	1.29
OBOX2 01	1.42	1.29
ZEC 01	1.28	1.41
SF1 Q6 01	1.28	1.34
NF-kappaB	1.28	1.28
NUR77 Q5	1.28	1.43
RORA Q4	1.34	1.28
Zfx	1.28	1.34
NKX21 01	1.27	1.33
E2F1DP1 01	1.27	1.33
MEF2 04	1.68	1.27
Mafb	1.31	1.27
RORA 1	1.27	1.27
AP1 C	1.27	1.26
PADS C	1.25	1.28
POLY C	1.25	1.36
FREAC2 01	1.32	1.25
ERR1 Q2	1.24	1.66
UF1H3BETA Q6	1.24	1.25
MTF1 02	1.33	1.24
EFC Q6	1.6	1.24
ER Q6	1.31	1.23
FOXF2	1.23	1.42
AP2GAMMA 01	1.22	1.24
GABPA	1.22	1.26
SP1 Q2 01	1.3	1.21
AP1 Q4 01	1.25	1.21
STAT3 03	1.21	1.54
CREB1	1.21	1.32
ZFP281 01	1.21	1.29
OBOX3 01	1.24	1.21
TEL2 Q6	1.21	1.25

CETS1P54 01	1.2	1.24
RFX Q6	1.24	1.2
SREBP2 Q6	1.2	1.25
KLF15 Q2	1.36	1.2
MIF1 01	1.2	1.23
TST1 01	1.37	1.2
RORA 2	1.2	1.33
AHR 01	1.2	1.37
MYB Q3	1.19	1.24
MAZR 01	1.23	1.19
MMEF2 Q6	1.56	1.19
SP1 Q4 01	1.19	1.23
ZF5 B	1.19	1.33
E2F1 Q6 01	1.29	1.18
EGR3 01	1.18	1.29
USF Q6	1.26	1.18
Egr1	1.19	1.18
AP1 01	1.18	1.38
IK2 01	1.21	1.18
P53 03	1.18	1.19
E2F1 Q3	1.18	1.28
ER Q6 02	1.21	1.18
HOXB8 01	1.18	1.29
AP2ALPHA 01	1.17	1.25
TFAP2A	1.17	1.25
IK1 01	1.17	1.18
WT1 Q6	1.38	1.17
P300 01	1.17	1.31
E2F1 Q4	1.22	1.17
PU1 Q6	1.17	1.18
E2F4DP2 01	1.17	1.26
Stat3	1.17	1.25
NFKB C	1.17	1.2
P53 01	1.17	1.42
ARNT 01	1.2	1.17
SP1 Q6 01	1.17	1.17
RUNX1	1.17	1.49
XVENT1 01	1.17	1.32
SRF	1.36	1.17
CBF 02	1.17	1.16
GATA1 04	1.16	1.27
AP1FJ Q2	1.16	1.4
SP1SP3 Q4	1.16	1.26
FREAC4 01	1.16	1.26
SF1 Q6	1.16	1.33
GR Q6 01	1.16	1.46
MECP2 02	1.17	1.16
SOX4 01	1.22	1.16
FEV	1.16	1.36
FRA1 Q5	1.25	1.16
AP4 Q6 01	1.19	1.16
PITX3 01	1.21	1.16
MAX	1.16	1.19
DAX1 01	1.22	1.15
SP1	1.3	1.15
CREB Q4	1.15	1.52
EGR2 01	1.15	1.2
FOXP1 01	0.84	1.86

CDX Q5	0.96	1.82
PXRRXR 02	0.86	1.78
HOXA9 01	0.65	1.62
EAR2 Q2	1.01	1.61
CREB Q2	1.08	1.6
DMRT7 01	0.91	1.54
NFAT Q4 01	0.62	1.53
MYOGNF1 01	1.07	1.52
FOXO1 Q5	0.86	1.48
AHR Q5	1.05	1.48
KAISO 01	0.93	1.48
ZNF219 01	1.1	1.48
IRF2 01	1.02	1.45
Spz1	1.05	1.43
OSF2 Q6	0.88	1.43
CDC5 01	0.69	1.41
DMRT3 01	0	1.41
PBX1 01	0.63	1.41
TBX15 02	0.61	1.4
HNF1B 01	1.02	1.4
CREBP1 Q2	1.04	1.39
FXR Q3	0.99	1.39
IRF7 01	1.04	1.39
Pax2	1.04	1.38
Pax5	1	1.38
PAX6 01	0.97	1.38
HOXB9 01	0.71	1.38
STAT1	1.07	1.38
CEBP C	0.98	1.37
AHRARNT 02	1.05	1.37
FOXO4 01	0.65	1.37
E2F1 Q6	1.07	1.36
GR 01	0.96	1.36
NFKAPPAB65 01	0.99	1.36
ATF1 Q6	0.98	1.34
CREB Q4 01	1.06	1.34
DMRT5 01	0.72	1.33
HOMEZ 01	0.97	1.33
PAX9 B	0.9	1.33
HBP1 Q2	0.77	1.33
INSM1	1.03	1.33
CREB Q2 01	0.98	1.33
PEBP Q6	0.78	1.33
IRF2	1.01	1.32
PITX2 Q2	1.02	1.32
AR 03	1.08	1.32
YY1 Q6 02	0.95	1.32
MAF Q6	1.06	1.32
IRF Q6 01	1.05	1.31
TITF1 Q3	0.88	1.31
GEN INI2 B	0.93	1.31
Nr2e3	0.91	1.3
ZTA Q2	1.06	1.3
DEAF1 01	0.95	1.29
GATA2 01	0.88	1.29
AFP1 Q6	0.52	1.29
CREB 02	0.87	1.29
STAT1 02	0.78	1.29

TAL1BETAE47 01	0.92	1.28
NFKB Q6	0.99	1.28
HNF1 02	0.64	1.28
NRF1 Q6	1.04	1.27
CBF 01	0.96	1.27
E12 Q6	0.74	1.27
RELA	1.04	1.27
GRE C	0.83	1.27
SP3 Q3	1.09	1.26
AML Q6	1.06	1.26
CP2 01	0.96	1.26
HNF4ALPHA Q6	0.97	1.26
CEBPGAMMA Q6	0.95	1.26
COUP 01	0.89	1.25
NR2F1	0.89	1.25
YY1 Q6	1.01	1.25
SRF Q5 02	0.9	1.25
PAX8 01	0.96	1.24
ATF 01	1.03	1.24
TAXCREB 01	0.74	1.24
SRF 01	0.97	1.23
MTF1 Q4	1.08	1.23
IPF1 Q4 01	0.67	1.23
CEBPA	0.89	1.23
CREBATF Q6	1.08	1.23
RNF96 01	1.06	1.23
PAX4 04	0.85	1.23
CMYB 01	1.08	1.22
HNF3A 01	0.94	1.22
ETS Q4	1.04	1.22
SZF11 01	1.05	1.22
ETS Q6	1.08	1.21
REST 01	1.01	1.21
STAT1 05	1	1.2
WHN B	1.09	1.2
ETS1 B	1.04	1.2
IK3 01	0.95	1.19
NKX25 03	0.68	1.19
AP2ALPHA 03	1.04	1.19
FOXO3	1	1.19
FPM315 01	1.07	1.19
AP2ALPHA 02	0.98	1.19
TCF4 Q5	0.66	1.18
LEF1 Q2 01	0.97	1.18
BARHL2 01	0.5	1.18
E4F1 Q6	0.9	1.18
PAX6 Q2	0.94	1.18
ZF5 01	0.86	1.18
LPOLYA B	0.9	1.18
NHLH1	1.04	1.18
ZBED6 01	1.08	1.17
PUR1 Q4	1.04	1.17
STAT6 01	1	1.17
HMBOX1 01	0.53	1.17
DLX7 01	0.95	1.17
FOXI1	0.96	1.17
GATA1 02	1.05	1.16
TCF3 01	0.78	1.16

TCF11 01	1.04	1.16
HNF3ALPHA Q6	0.96	1.16
IRF1 01	1.04	1.16
SRF 02	1.08	1.16
HIC1 03	0.96	1.16
AP1 Q4	1.07	1.16
HNF3 Q6	1	1.16
NURR1 Q3	1.07	1.15
SEF1 C	1.03	1.15
En1	0.8	1.15
PAX6 02	1.66	0
BRCA 01	1.57	0.75
HEB Q6	1.54	0.95
CART1 02	1.51	0
XFD1 01	1.5	0
HMGIY 01	1.5	0.9
SOX9 Q4	1.49	1.04
GATA1 05	1.49	0.94
CART1 03	1.49	0
ACAAT B	1.45	0.94
EN1 02	1.44	0
PAX7 01	1.43	0
GATA Q6	1.42	0.89
RBPJK Q4	1.41	0.97
ESR1	1.41	1.07
TFE Q6	1.4	1.08
HOXD3 01	1.39	1.03
RFX1 02	1.37	1.02
GATA2 03	1.36	0.56
ATF6 01	1.36	1.08
PPARG 02	1.36	0.77
USF 01	1.36	1.08
KROX Q6	1.35	1.04
AP4 Q5	1.34	1.02
NF1 Q6 01	1.34	1.01
NR3C1	1.33	1.09
NANOG 02	1.33	0.88
E2F1DP2 01	1.33	1.01
SMAD1 01	1.33	0.94
CDP 03	1.32	0.9
TCF11MAFG 01	1.32	0.89
HNF4 01 B	1.31	1.05
E2F 02	1.31	1.07
LXR DR4 Q3	1.3	1.04
SPIB 01	1.3	0.98
TBX5 01	1.3	1.03
CDP 04	1.29	0.88
FREAC7 01	1.29	0
IPF1 03	1.29	0.98
PPARG	1.28	1
GATA2 02	1.28	0.87
RREB1 01	1.28	1.02
T3R 01	1.28	0.96
GAF Q6	1.28	0.93
DOBOX4 01	1.28	0.66
MOX1 01	1.28	0.63
PAX4 02	1.28	0.69
HOXC4 01	1.28	0

GFI1B 01	1.28	0.89
PAX4 03	1.27	0.99
TATA 01	1.27	0.88
TBP	1.27	0.88
MEF3 B	1.26	0.97
HOXA3 02	1.26	1.06
PMX2B 01	1.26	0.65
HOXC5 01	1.26	0
ALX4 02	1.26	0.53
GATA3 02	1.26	0.79
PARP Q3	1.26	0.93
Foxd3	1.26	0.76
Sox2	1.25	1.04
TEF 01	1.25	0.98
TEAD1	1.25	0.98
