# Supplementary material for: Genome-Wide Analysis of Functional and Evolutionary Features of Tele-Enhancers
Source: G3 (Bethesda). 2014 Feb 4;4(4):579–93. doi: 10.1534/g3.114.010447 (PMC4059231; doi:10.1534/g3.114.010447)
Supplement: Supporting Information [file supp_g3.114.010447_TableS6.pdf]

Table S6 Nucleotide divergence and SNP distribution of intergenic/intronic *tele* and proximal heart enhancers (a) the value of divergence and SNP density; (b) p-values for all comparisons.

(a)

	divergence per 1000 bp		NI	total divergence		#SNP per 1000bp	Fraction of low DAF
	human	non-human					
Intronic							
tele	5.2995	58.7827	0.73	12889	142965	5.34	0.5432
proximal	5.4781	58.1271	0.77	7650	81173	5.41	0.5363
Intergenic							
tele	5.8521	61.1104	0.77	6355	66362	5.63	0.5238
proximal	5.882	62.2086	0.78	6818	72108	5.92	0.5273

(b)

NI			
	tele	proximal	pvalue
Intronic	0.73	0.77	1.00E-02
Intergenic	0.77	0.78	0.75
pvalue	1.00E-03	0.86	
SNP density			
	tele	proximal	pvalue
Intronic	5.34	5.41	3.00E-02
Intergenic	5.63	5.92	9.00E-06
pvalue	5.00E-04	8.00E-16	
Fraction of low DAF			
	tele	proximal	pvalue
Intronic	0.5432	0.5363	1.00E-01
Intergenic	0.5238	0.5273	0.43
Pvalue	5.00E-03	0.07	
