# Supplementary material for: Genome-Wide Analysis of Functional and Evolutionary Features of Tele-Enhancers
Source: G3 (Bethesda). 2014 Feb 4;4(4):579–93. doi: 10.1534/g3.114.010447 (PMC4059231; doi:10.1534/g3.114.010447)
Supplement: Supporting Information [file supp_g3.114.010447_TableS7.pdf]

Table S7 GO biological processes associated with *tele* and proximal brain enhancers.

GOID	GO	#genes	Proximal			Tele		
			#genes	Enrichment Fold	p-value	#genes	Enrichment Fold	p-value
GO:0030900	forebrain development	228	135	14.21	0.00E+00	78	9.048112	0.00E+00
GO:0021537	telencephalon development	135	83	15.2	0.00E+00	47	11.683002	0.00E+00
GO:0031175	neurite development	471	148	5.42	0.00E+00	80	3.663203	0.00E+00
GO:0007423	sensory organ development	326	76	3.8	0.00E+00	63	3.390351	0.00E+00
GO:0048812	neurite morphogenesis	420	140	5.77	0.00E+00	69	3.638227	0.00E+00
GO:0009792	embryonic development ending in birth or egg hatching	406	79	2.92	0.00E+00	71	3.33896	0.00E+00
GO:0030182	neuron differentiation	658	209	5.44	0.00E+00	114	3.521227	0.00E+00
GO:0007417	central nervous system development	558	239	8.04	0.00E+00	183	8.763962	0.00E+00
GO:0021543	pallium development	90	51	16.82	0.00E+00	30	11.185853	0.00E+00
GO:0009790	embryonic development	655	123	2.67	0.00E+00	104	2.76983	0.00E+00
GO:0021953	central nervous system neuron differentiation	93	58	13.04	0.00E+00	27	9.396117	0.00E+00
GO:0048646	anatomical structure formation	518	103	3.31	0.00E+00	83	3.282313	0.00E+00
GO:0051960	regulation of nervous system development	345	110	5.61	0.00E+00	65	4.712558	0.00E+00
GO:0022037	metencephalon development	64	39	17.53	0.00E+00	24	12.528155	0.00E+00
GO:0007420	brain development	409	213	11.09	0.00E+00	166	11.709875	0.00E+00
GO:0030902	hindbrain development	94	54	12.72	0.00E+00	36	12.528155	0.00E+00
GO:0000904	cellular morphogenesis during differentiation	478	154	5.33	0.00E+00	77	3.19004	0.00E+00
GO:0032989	cellular structure morphogenesis	634	175	4.39	0.00E+00	95	2.817649	0.00E+00
GO:0003002	regionalization	215	62	4.65	0.00E+00	52	5.121573	0.00E+00
GO:0048598	embryonic morphogenesis	345	84	3.78	0.00E+00	63	3.57461	0.00E+00
GO:0007409	axonogenesis	387	127	5.66	0.00E+00	69	3.915049	0.00E+00
GO:0050767	regulation of neurogenesis	313	101	5.68	0.00E+00	55	4.222111	0.00E+00
GO:0021549	cerebellum development	56	33	16.32	0.00E+00	21	15.660194	0.00E+00
GO:0048667	neuron morphogenesis during differentiation	410	137	5.55	0.00E+00	70	3.690955	0.00E+00
GO:0048666	neuron development	556	173	5.31	0.00E+00	89	3.182094	0.00E+00
GO:0045595	regulation of cell differentiation	765	149	3.36	0.00E+00	104	2.728074	0.00E+00
GO:0021915	neural tube development	94	38	8.95	0.00E+00	30	6.80878	4.44E-13
GO:0045664	regulation of neuron differentiation	261	86	5.91	0.00E+00	44	4.253386	3.11E-12
GO:0021987	cerebral cortex development	62	35	17.31	0.00E+00	19	11.020137	1.47E-11
GO:0035295	tube development	326	71	3.38	0.00E+00	54	3.396187	2.84E-11
GO:0021766	hippocampus development	44	25	20.61	0.00E+00	17	12.6773	2.98E-11
GO:0009887	organ morphogenesis	597	115	3.11	0.00E+00	81	2.578203	4.22E-11

GO:0022603	regulation of anatomical structure morphogenesis	440	94	3.34	0.00E+00	68	2.862616	4.62E-11
GO:0031399	regulation of protein modification process	831	109	2.2	5.77E-11	97	2.322689	5.95E-11
GO:0048585	negative regulation of response to stimulus	563	90	2.5	9.77E-12	75	2.645303	1.13E-10
GO:0009953	dorsal/ventral pattern formation	68	23	8.12	2.44E-11	23	7.503843	1.29E-10
GO:0009725	response to hormone stimulus	656	91	2.43	3.42E-11	76	2.592973	2.08E-10
GO:0051093	negative regulation of developmental process	401	80	3.6	0.00E+00	61	2.921321	4.24E-10
GO:0021536	diencephalon development	54	30	10.6	0.00E+00	21	7.830097	6.05E-10
GO:0006928	cell motility	701	117	2.72	0.00E+00	82	2.418335	8.41E-10
GO:0021872	generation of neurons in the forebrain	25	13	16.07	1.02E-09	12	20.880259	2.60E-10
GO:0021795	cerebral cortex cell migration	25	14	69.24	0.00E+00	8	41.760518	3.11E-09
GO:0016477	cell migration	483	89	3.14	0.00E+00	60	2.796463	4.07E-09
GO:0007267	cell-cell signaling	737	142	3.14	0.00E+00	84	2.307818	4.90E-09
GO:0051094	positive regulation of developmental process	535	97	3.12	0.00E+00	65	2.650814	5.09E-09
GO:0048854	brain morphogenesis	18	9	44.51	1.00E-10	12	15.660194	9.20E-09
GO:0051129	negative regulation of cellular component organization and biogenesis	256	49	3.15	7.50E-09	40	3.728618	3.82E-09
GO:0048568	embryonic organ development	241	53	2.95	1.25E-08	52	4.308625	0.00E+00
GO:0008283	cell proliferation	480	74	2.77	1.87E-11	61	2.675831	1.64E-08
GO:0009952	anterior/posterior pattern formation	150	36	3.87	1.66E-08	41	6.294784	0.00E+00
GO:0045165	cell fate commitment	156	43	3.94	1.01E-10	31	4.373568	1.91E-08
GO:0008284	positive regulation of cell proliferation	529	85	2.82	0.00E+00	63	2.610032	2.11E-08
GO:0031327	negative regulation of cellular biosynthetic process	758	131	2.99	0.00E+00	86	2.200616	3.09E-08
GO:0040007	growth	267	49	3.07	1.85E-08	41	3.508568	1.39E-08
GO:0016265	death	803	104	2.09	5.08E-09	90	2.155073	3.03E-08
GO:0008219	cell death	800	103	2.08	9.04E-09	90	2.155073	3.03E-08
GO:0030111	regulation of Wnt receptor signaling pathway	127	31	4.51	9.08E-09	25	5.220065	3.84E-08
GO:0007610	behavior	356	86	4.21	0.00E+00	49	2.974223	5.08E-08
GO:0001932	regulation of protein amino acid phosphorylation	678	91	2.26	2.03E-09	77	2.283778	6.83E-08
GO:0048870	cell motility involved in cell locomotion	520	93	2.99	0.00E+00	62	2.548378	8.12E-08
GO:0051674	localization of cell	520	93	2.99	0.00E+00	62	2.548378	8.12E-08
GO:0045596	negative regulation of cell differentiation	312	66	3.67	0.00E+00	49	2.906627	1.12E-07
GO:0021954	central nervous system neuron development	42	26	12.86	0.00E+00	10	17.400216	1.27E-07

GO:0021695	cerebellar cortex development	30	19	23.49	0.00E+00	12	12.528155	1.41E-07
GO:0021575	hindbrain morphogenesis	28	19	23.49	0.00E+00	11	14.355178	1.59E-07
GO:0009968	negative regulation of signal transduction	459	72	2.41	3.34E-08	59	2.588099	1.32E-07
GO:0009890	negative regulation of biosynthetic process	771	131	2.94	0.00E+00	86	2.127609	1.67E-07
GO:0031344	regulation of cell projection organization and biogenesis	202	54	5.68	0.00E+00	32	3.884699	1.92E-07
GO:0035270	endocrine system development	115	35	4.33	1.40E-09	23	5.220065	2.23E-07
GO:0043069	negative regulation of programmed cell death	505	85	3.14	0.00E+00	60	2.525838	2.36E-07
GO:0043066	negative regulation of apoptosis	500	84	3.12	0.00E+00	60	2.525838	2.36E-07
GO:0051130	positive regulation of cellular component organization and biogenesis	370	67	3.28	0.00E+00	52	2.741852	2.38E-07
GO:0048562	embryonic organ morphogenesis	155	38	3.36	2.89E-07	40	5.643313	0.00E+00
GO:0045934	negative regulation of nucleobase, nucleoside, nucleotide and nucleic acid metabolic process	700	123	3.01	0.00E+00	78	2.189059	3.64E-07
GO:0051253	negative regulation of RNA metabolic process	611	107	3.02	0.00E+00	71	2.287806	3.78E-07
GO:0045597	positive regulation of cell differentiation	401	76	3.45	0.00E+00	50	2.747402	5.00E-07
GO:0042493	response to drug	342	49	2.79	5.03E-07	50	3.182966	2.83E-09
GO:0048839	inner ear development	116	32	4.4	8.26E-09	28	4.176052	5.05E-07
GO:0001654	eye development	211	49	3.85	4.88E-12	38	3.251844	6.75E-07
GO:0051172	negative regulation of nitrogen compound metabolic process	710	124	2.99	0.00E+00	78	2.154312	7.54E-07
GO:0045892	negative regulation of transcription, DNA-dependent	589	106	3.08	0.00E+00	69	2.279649	7.95E-07
GO:0042325	regulation of phosphorylation	740	101	2.32	2.04E-11	81	2.114126	8.21E-07
GO:0009991	response to extracellular stimulus	344	54	2.59	8.44E-07	52	3.270402	3.66E-10
GO:0016055	Wnt receptor signaling pathway	139	30	3.8	1.19E-06	33	4.306553	6.59E-09
GO:0045944	positive regulation of transcription from RNA polymerase II promoter	503	107	3.31	0.00E+00	59	2.425069	1.57E-06
GO:0019220	regulation of phosphate metabolic process	792	109	2.29	4.00E-12	86	2.031337	1.59E-06
GO:0051174	regulation of phosphorus metabolic process	792	109	2.29	4.00E-12	86	2.031337	1.59E-06
GO:0043583	ear development	132	38	4.7	1.20E-11	29	3.784547	2.45E-06
GO:0016331	morphogenesis of embryonic epithelium	87	35	8.24	0.00E+00	23	4.61775	2.48E-06
GO:0021587	cerebellum morphogenesis	26	18	29.67	0.00E+00	10	13.050162	2.53E-06

GO:0035148	lumen formation	77	30	7.81	0.00E+00	21	4.982789	3.03E-06
GO:0040008	regulation of growth	421	63	2.29	4.15E-06	58	3.186987	4.53E-11
GO:0051050	positive regulation of transport	370	57	2.68	7.51E-08	48	2.637506	4.21E-06
GO:0048729	tissue morphogenesis	326	68	3.2	4.44E-13	47	2.638097	6.12E-06
GO:0021772	olfactory bulb development	20	12	14.84	1.80E-08	6	31.320388	6.22E-06
GO:0021988	olfactory lobe development	20	12	14.84	1.80E-08	6	31.320388	6.22E-06
GO:0007411	axon guidance	300	95	5.22	0.00E+00	42	2.810804	6.66E-06
GO:0060070	Wnt receptor signaling pathway through beta-catenin	64	18	5.56	6.70E-06	23	7.062441	4.61E-10
GO:0033273	response to vitamin	148	30	3.53	6.70E-06	27	4.698058	7.38E-08
GO:0008285	negative regulation of cell proliferation	433	74	2.67	1.18E-10	48	2.583125	8.17E-06
GO:0007584	response to nutrient	221	38	2.94	1.16E-05	34	3.549644	5.70E-07
GO:0001505	regulation of neurotransmitter levels	89	20	4.95	8.12E-06	21	4.766146	6.73E-06
GO:0048521	negative regulation of behavior	20	6	29.67	9.02E-06	6	31.320388	6.22E-06
GO:0031346	positive regulation of cell projection organization and biogenesis	105	23	4.95	6.63E-07	18	5.220065	1.82E-05
GO:0022029	telencephalon cell migration	32	21	25.96	0.00E+00	10	10.440129	2.47E-05
GO:0012501	programmed cell death	677	83	1.95	2.49E-05	76	2.179807	7.59E-07
GO:0007268	synaptic transmission	467	112	4.47	0.00E+00	53	2.364645	2.61E-05
GO:0030901	midbrain development	22	11	9.07	2.64E-05	13	13.572168	9.44E-09
GO:0043549	regulation of kinase activity	543	74	2.38	3.07E-08	60	2.221304	2.85E-05
GO:0019226	transmission of nerve impulse	527	131	4.8	0.00E+00	59	2.231767	3.19E-05
GO:0051338	regulation of transferase activity	554	75	2.36	2.93E-08	61	2.196027	3.27E-05
GO:0001838	embryonic epithelial tube formation	71	29	8.44	0.00E+00	19	4.722916	3.92E-05
GO:0021846	cell proliferation in forebrain	19	12	59.35	0.00E+00	8	13.920173	4.35E-05
GO:0001841	neural tube formation	59	24	9.13	4.44E-13	17	5.220065	4.40E-05
GO:0031667	response to nutrient levels	321	48	2.45	4.60E-05	50	3.676102	1.29E-11
GO:0021879	forebrain neuron differentiation	17	10	12.36	4.43E-06	8	13.920173	4.35E-05
GO:0045859	regulation of protein kinase activity	518	70	2.32	2.90E-07	57	2.237171	5.13E-05
GO:0035239	tube morphogenesis	211	51	3.71	5.33E-12	34	2.958037	5.60E-05
GO:0001944	vasculature development	343	51	2.36	5.67E-05	46	3.039531	9.93E-08
GO:0048589	developmental growth	159	36	3.96	8.90E-09	25	3.625045	7.05E-05
GO:0021904	dorsoventral neural tube patterning	15	8	13.19	6.90E-05	6	31.320388	6.22E-06
GO:0048545	response to steroid hormone stimulus	324	49	2.72	1.07E-06	39	2.678717	8.47E-05
GO:0014020	primary neural tube	50	21	8.65	8.66E-11	14	6.090076	8.73E-05

	formation							
GO:0042471	ear morphogenesis	74	20	4.3	8.69E-05	22	5.220065	5.39E-07
GO:0050769	positive regulation of neurogenesis	102	30	4.79	4.40E-09	18	4.698058	9.47E-05
GO:0009798	axis specification	64	18	4.69	1.00E-04	18	5.872573	2.77E-06
GO:0001764	neuron migration	77	30	7.81	0.00E+00	16	4.395844	1.16E-03
GO:0048863	stem cell differentiation	77	32	6.88	0.00E+00	15	4.605939	1.34E-03
GO:0016192	vesicle-mediated transport	680	85	2.03	2.28E-06	75	1.80417	2.17E-03
GO:0050770	regulation of axonogenesis	84	28	6.29	2.93E-11	15	4.350054	2.80E-03
GO:0007167	enzyme linked receptor protein signaling pathway	583	85	2.3	5.29E-09	56	1.988596	2.98E-03
GO:0001843	neural tube closure	48	21	8.65	8.66E-11	12	5.220065	3.64E-03
GO:0031345	negative regulation of cell projection organization and biogenesis	63	20	6.18	1.59E-07	12	5.220065	3.64E-03
GO:0051969	regulation of transmission of nerve impulse	176	53	7.28	0.00E+00	24	2.982894	4.45E-03
GO:0022604	regulation of cell morphogenesis	222	58	4.16	0.00E+00	33	2.460888	5.58E-03
GO:0043523	regulation of neuron apoptosis	138	34	4.54	7.43E-10	22	3.103822	5.82E-03
GO:0045665	negative regulation of neuron differentiation	42	14	6.92	1.66E-05	10	5.800072	7.99E-03
GO:0007611	learning and/or memory	141	41	6.54	0.00E+00	21	3.132039	8.20E-03
GO:0021515	cell differentiation in spinal cord	31	16	9.89	7.81E-09	8	6.960086	1.34E-02
GO:0050804	regulation of synaptic transmission	162	48	7.42	0.00E+00	22	2.944652	1.37E-02
GO:0006836	neurotransmitter transport	91	20	4.3	8.69E-05	18	3.355756	1.38E-02
GO:0021696	cerebellar cortex morphogenesis	19	14	69.24	0.00E+00	6	7.830097	6.21E-02
GO:0021895	cerebral cortex neuron differentiation	12	9	44.51	1.00E-10	5	8.700108	1.21E-01
GO:0021680	cerebellar Purkinje cell layer development	17	10	49.46	1.78E-12	5	8.700108	1.21E-01
GO:0021955	central nervous system neuron axonogenesis	21	14	17.31	5.42E-11	4	20.880259	1.00E+00
GO:0046907	intracellular transport	827	79	1.58	1.36E-01	111	2.41428	0.00E+00
GO:0021854	hypothalamus development	14	5	6.18	7.73E-01	9	46.980583	5.86E-11
GO:0046903	secretion	375	48	2.01	1.26E-02	55	2.84261	1.81E-08
GO:0035282	segmentation	56	15	3.9	1.11E-02	18	6.264078	9.72E-07
GO:0010243	response to organic nitrogen	134	25	3.02	2.35E-03	24	4.640058	1.03E-06
GO:0042472	inner ear morphogenesis	64	16	3.96	4.85E-03	20	5.494805	1.27E-06
GO:0031099	regeneration	109	17	2.9	1.60E-01	21	4.982789	3.03E-06
GO:0032107	regulation of response to nutrient levels	23	0	0	1.00E+00	6	31.320388	6.22E-06
GO:0032104	regulation of response to extracellular stimulus	23	0	0	1.00E+00	6	31.320388	6.22E-06
GO:0017145	stem cell division	17	6	7.42	8.75E-02	6	31.320388	6.22E-06

GO:0016568	chromatin modification	336	41	1.73	1.00E+00	45	2.700033	6.48E-06
GO:0032940	secretion by cell	299	42	2.16	8.04E-03	43	2.771145	6.66E-06
GO:0003001	generation of a signal involved in cell-cell signaling	111	23	3.07	4.28E-03	21	4.766146	6.73E-06
GO:0031329	regulation of cellular catabolic process	409	57	1.89	1.15E-02	51	2.511541	6.92E-06
GO:0051169	nuclear transport	237	20	1.71	1.00E+00	36	3.080694	7.93E-06
GO:0006913	nucleocytoplasmic transport	235	20	1.77	1.00E+00	36	3.080694	7.93E-06
GO:0048704	embryonic skeletal morphogenesis	57	15	3.09	1.85E-01	14	7.308091	8.05E-06
GO:0051090	regulation of transcription factor activity	254	32	1.72	1.00E+00	39	2.908322	9.27E-06
GO:0051247	positive regulation of protein metabolic process	469	51	1.84	7.22E-02	53	2.405769	1.47E-05
GO:0051276	chromosome organization and biogenesis	528	53	1.6	1.00E+00	61	2.242422	1.53E-05
GO:0033043	regulation of organelle organization and biogenesis	357	46	2.01	1.91E-02	52	2.423601	1.60E-05
GO:0000278	mitotic cell cycle	507	40	1.39	1.00E+00	60	2.253265	1.71E-05
GO:0045787	positive regulation of cell cycle	94	12	1.98	1.00E+00	19	4.959061	1.77E-05
GO:0000086	G2/M transition of mitotic cell cycle	112	11	2.09	1.00E+00	22	4.253386	2.46E-05
GO:0032270	positive regulation of cellular protein metabolic process	413	48	1.99	1.57E-02	49	2.459454	2.69E-05
GO:0010035	response to inorganic substance	336	43	2.31	1.12E-03	44	2.580706	3.63E-05
GO:0045740	positive regulation of DNA replication	37	6	2.7	1.00E+00	9	11.745146	3.69E-05
GO:0006886	intracellular protein transport	513	51	1.74	3.02E-01	62	2.157627	4.73E-05
GO:0001558	regulation of cell growth	242	33	2.12	1.21E-01	33	3.022143	5.30E-05
GO:0051329	interphase of mitotic cell cycle	293	23	1.5	1.00E+00	38	2.755034	6.03E-05
GO:0001701	in utero embryonic development	259	35	1.94	4.10E-01	37	2.799165	6.03E-05
GO:0051704	multi-organism process	830	78	1.58	1.55E-01	85	1.880108	6.77E-05
GO:0006325	establishment and/or maintenance of chromatin architecture	404	48	1.84	1.23E-01	48	2.409261	7.07E-05
GO:0051325	interphase	305	23	1.44	1.00E+00	38	2.717294	8.63E-05
