# Supplementary material for: Genome-Wide Analysis of Functional and Evolutionary Features of Tele-Enhancers
Source: G3 (Bethesda). 2014 Feb 4;4(4):579–93. doi: 10.1534/g3.114.010447 (PMC4059231; doi:10.1534/g3.114.010447)
Supplement: Supporting Information [file supp_g3.114.010447_TableS8.pdf]

Table S8 GO biological processes associated with proximal and *tele* lung enhancers

GOID	GO	#genes	Proximal			Tele		
			#genes	Enrichment Fold	p-value	#genes	Enrichment Fold	p-value
GO:0030323	respiratory tube development	127	63	14.47	0.00E+00	54	14.351829	0.00E+00
GO:0009725	response to hormone stimulus	656	94	2.97	0.00E+00	81	3.146362	0.00E+00
GO:0035295	tube development	326	90	6.02	0.00E+00	69	4.907472	0.00E+00
GO:0009719	response to endogenous stimulus	805	116	2.85	0.00E+00	94	2.912107	0.00E+00
GO:0009611	response to wounding	817	119	2.83	0.00E+00	102	2.488267	0.00E+00
GO:0030324	lung development	123	63	15.12	0.00E+00	52	14.588073	0.00E+00
GO:0009967	positive regulation of signal transduction	603	103	3.26	0.00E+00	78	2.773788	2.66E-12
GO:0008284	positive regulation of cell proliferation	529	89	3.46	0.00E+00	69	2.927987	9.33E-12
GO:0043068	positive regulation of programmed cell death	504	67	2.97	1.20E-11	71	2.86824	1.02E-11
GO:0043065	positive regulation of apoptosis	500	67	2.97	1.20E-11	71	2.86824	1.02E-11
GO:0014070	response to organic cyclic substance	222	35	3.24	3.42E-06	25	2.744412	1.24E-02
GO:0048598	embryonic morphogenesis	345	62	3.48	0.00E+00	39	2.188211	1.28E-02
GO:0045596	negative regulation of cell differentiation	312	69	4.34	0.00E+00	34	2.32014	1.42E-02
GO:0035239	tube morphogenesis	211	46	4.34	4.44E-13	26	2.431345	7.02E-02
GO:0030855	epithelial cell differentiation	180	40	3.99	4.69E-10	28	2.524859	1.82E-02
GO:0008283	cell proliferation	480	69	2.6	2.41E-09	48	1.970621	2.06E-02
GO:0030029	actin filament-based process	274	51	3.64	1.02E-11	32	2.341898	2.14E-02
GO:0033157	regulation of intracellular protein transport	116	23	4.5	4.09E-06	16	3.512847	2.26E-02
GO:0012502	induction of programmed cell death	322	43	3.11	1.99E-07	38	2.156059	2.34E-02
GO:0055093	response to hyperoxia	21	5	3.77	1.00E+00	10	16.832392	1.79E-07
GO:0007585	respiratory gaseous exchange	36	5	2.2	1.00E+00	12	12.119322	2.09E-07
GO:0055082	cellular chemical homeostasis	448	45	1.92	7.66E-02	51	2.627914	1.48E-06
GO:0046039	GTP metabolic process	213	19	1.45	1.00E+00	38	3.094988	2.62E-06
GO:0006184	GTP catabolic process	205	18	1.44	1.00E+00	37	3.113992	3.61E-06
GO:0045730	respiratory burst	15	0	0	1.00E+00	6	30.298305	7.80E-06
