# Supplementary material for: Genome-Wide Analysis of Functional and Evolutionary Features of Tele-Enhancers
Source: G3 (Bethesda). 2014 Feb 4;4(4):579–93. doi: 10.1534/g3.114.010447 (PMC4059231; doi:10.1534/g3.114.010447)
Supplement: Supporting Information [file supp_g3.114.010447_TableS9.pdf]

Table S9 Nucleotide divergence (per kilobase) of *tele* and proximal enhancers in cell types.

cell	proximal			tele			p-value (tele vs proximal)
	Non-human	human	NI	Non-human	human	NI	
GM12878	59.1112	5.3352	0.74	59.7612	5.2197	0.71	5.98E-06
H1-Hesc	56.4927	5.5512	0.8	58.2957	5.3274	0.75	6.03E-08
HepG2	58.1545	5.4365	0.76	59.4398	5.3354	0.73	6.83E-07
HSMM	54.7468	5.2121	0.78	56.553	5.254	0.76	2.13E-03
HUVEC	55.904	5.3227	0.78	57.6571	5.2051	0.74	2.36E-11
K562	58.6319	5.4612	0.76	60.7164	5.4047	0.73	6.98E-08
NHEK	56.6584	5.2833	0.76	58.1341	5.1209	0.72	1.97E-15
